# Functional MRI signals exhibit stronger covariation with peripheral autonomic measures as vigilance decreases

**DOI:** 10.1162/imag_a_00287

**Published:** 2024-09-13

**Authors:** Benjamin P. Gold, Sarah E. Goodale, Chong Zhao, Haatef Pourmotabbed, Jacco A. de Zwart, Pinar S. Özbay, Taylor S. Bolt, Jeff H. Duyn, Jingyuan E. Chen, Catie Chang

**Affiliations:** Department of Electrical and Computer Engineering, Vanderbilt University, Nashville, TN, United States; Vanderbilt University Institute of Imaging Science, Vanderbilt University Medical Center, Nashville, TN, United States; Department of Biomedical Engineering, Vanderbilt University, Nashville, TN, United States; Advanced MRI Section, Laboratory of Functional and Molecular Imaging, National Institute of Neurological Disorders and Stroke, National Institutes of Health, Bethesda, MD, United States; Institute of Biomedical Engineering, Bogazici University, Istanbul, Turkey; Department of Psychiatry and Biobehavioral Sciences, University of California, Los Angeles, Los Angeles, CA, United States; Athinoula A. Martinos Center for Biomedical Imaging, Massachusetts General Hospital, Boston, MA, United States; Department of Radiology, Harvard Medical School, Boston, MA, United States; Department of Computer Science, Vanderbilt University, Nashville, TN, United States

**Keywords:** fMRI, vigilance, physiology, functional connectivity

## Abstract

Vigilance naturally drifts over time, coinciding with marked changes in brain-wide functional magnetic resonance imaging (fMRI) signals. Though the precise origins of these hemodynamic changes are unclear, largely separate lines of research have linked different vigilance levels not only to changes in fMRI signal fluctuation amplitudes and functional connectivity, but also to significant variations in autonomic physiology. These findings raise the possibility that vigilance-related modulations in fMRI signals may arise in part from changes in autonomic physiology and their effects on cerebral hemodynamics. Here, using simultaneous recordings of fMRI, EEG-indexed vigilance, respiration, and pulse oximetry, we investigate how the relationship between autonomic and fMRI signals varies systematically as vigilance gradually drifts. Regression analyses indicated that the strength and extent of fMRI-autonomic covariation increased as vigilance diminished, during both resting state and an auditory vigilance task. Spatiotemporally, autonomic signals exhibited early positive correlations and delayed negative correlations with fMRI signals throughout much of the grey matter, accompanied by late positive correlations in the ventricles and periventricular white matter. Low-frequency EEG power fluctuations also demonstrated state-dependent associations with both fMRI and autonomic signals, with effects in fMRI that partially overlapped with those of peripheral autonomic variations. Functional connectivity between most brain networks strengthened as vigilance decreased, especially during resting-state scans, and removing autonomic variance from fMRI signals largely attenuated this effect. Together, these results demonstrate interactions between vigilance levels, autonomic physiology, and brain hemodynamics, showing that the physiological constituents of fMRI signals vary markedly over vigilance levels and brain regions. These findings contribute to knowledge of human brain physiology and toward the accurate parsing, analysis, and interpretation of fMRI data.

## 1 Introduction

Functional magnetic resonance imaging (fMRI) is a widely used and powerful tool for understanding neural processes. Based on the magnetic susceptibility contrast between oxygenated and deoxygenated hemoglobin, this technique measures cerebral blood oxygen changes that are coupled with changes in neuronal activity (Bandettini et al., 1992;Kwong et al., 1992;Ogawa et al., 1990). Yet blood oxygen levels may also change for reasons not directly related to local neural activity, such as with systemic physiological effects like breathing-induced modulations of blood flow (Birn et al., 2006;Das et al., 2021;Kastrup et al., 1998;Wise et al., 2004). As a result, distinguishing regional neurovascular coupling from other, more global physiological processes is of central importance in fMRI analysis and neuroscience research (Iacovella & Hasson, 2011;T. T. Liu, 2016;Power et al., 2017;Xifra-Porxas et al., 2021). Importantly, systemic physiological components of fMRI do not merely present a confound, as they can provide valuable avenues for interrogating cerebrovascular health (Donahue et al., 2016;Makedonov et al., 2013) as well as interactions between brain function and brain-body physiology (Bright et al., 2020;Mather & Thayer, 2018;Yang et al., 2022).

Of the various phenomena that affect fMRI signals, the activities of the heart and lungs are among the most influential (Iacovella & Hasson, 2011;T. T. Liu, 2016). Some of these physiological processes have characteristic effects that are largely distinct from the fMRI signals generated by neural activity, like pulsations synchronized to the cardiac cycle that are most prominent near large blood vessels (Dagli et al., 1999;Glover et al., 2000). Others—like low-frequency (i.e., <0.1 Hz) fluctuations in heart rate, pulse amplitude, and breathing depth/rate—have spatial and temporal characteristics that are similar to those of blood oxygen level-dependent (BOLD) responses arising from local neurovascular coupling (Birn et al., 2006;Özbay et al., 2019;Shmueli et al., 2007;Wise et al., 2004).Physiologically linked fluctuations may also originate in the central nervous system (Cechetto, 2014;Dampney, 2016), such that attempts to remove their effects from fMRI signals could actually obscure neural activity linked with these autonomic processes (Duyn et al., 2020). These slow autonomic fluctuations can account for substantial variance in fMRI signals and the correlations between regions (a common method for inferring functional connectivity) (Chen et al., 2020;Xifra-Porxas et al., 2021).

A largely separate line of work has reported that fMRI variance is additionally related to fluctuations in EEG- and pupil-based measures of vigilance (here, referring to one’s level of wakefulness). Sleep is common in resting-state scans, with about one-third of participants reportedly falling asleep within 3 min and up to 65% doing so within 20 min (Soon et al., 2021;Tagliazucchi & Laufs, 2014). Along the continuum from alertness to light sleep, vigilance decrements are characterized by dramatic changes in cognition, behavior, and neural activity, as indicated by reduced oddball detection, decreased reaction times, and a shift in the power of neural oscillations from alpha (~8 - 12 Hz) to delta (~0.5 - 4 Hz) and theta (~3 - 7 Hz) rhythms (Merica & Fortune, 2004;Ogilvie, 2001;Wong et al., 2013). Meanwhile, spontaneous fMRI signals measured at rest become increasingly variable throughout much of the cortex and thalamus (Fukunaga et al., 2006;Horovitz et al., 2008;Larson-Prior et al., 2009). Even when participants have tasks to perform, making them less likely to sleep, decreases in vigilance coincide with significantly greater temporal variability in fMRI signals (Roth et al., 2020) and changes in functional connectivity (Wang et al., 2016). These vigilance-related effects are comparable in magnitude to those of task-induced fMRI responses (Fukunaga et al., 2006;Horovitz et al., 2008) and are thus capable of overshadowing other sources of fMRI variance during both rest and tasks.

Fluctuations in vigilance and autonomic activity tend to be highly correlated (Özbay et al., 2019;Shams et al., 2021;Yuan et al., 2013), and the descent into sleep coincides with reductions in heart rate, breathing rate, and blood pressure (Ogilvie, 2001;Silvani, 2008;Trinder et al., 1992,^2001^). These vigilance-coupled autonomic changes are accompanied by neural modulation in the hypothalamus, thalamus, basal forebrain, and brainstem, each of which form part of the circuits implicated in both arousal and autonomic physiology (Benarroch, 2017;Dampney, 2016;Duyn et al., 2020;X. Liu et al., 2018;Merica & Fortune, 2004;Silvani et al., 2015;Yackle et al., 2017). Consistent with these overlapping neural substrates, vigilance and autonomic activity also have remarkably overlapping effects on fMRI measurements (Gu et al., 2022;Picchioni et al., 2022;Raut et al., 2021;Soon et al., 2021): in particular, they covary with widespread changes in fMRI signals throughout much of the grey matter—especially in the subcortex, default mode, and salience networks—and in the ventricles and periventricular white matter (Gu et al., 2022;Özbay et al., 2019;Picchioni et al., 2022;Yuan et al., 2013). Likewise, regressing out peripheral autonomic fluctuations from fMRI results in significantly weaker vigilance effects, demonstrating that these phenomena share at least some portion of fMRI variance in addition to their shared neuroregulatory circuits (Duyn et al., 2020;Goodale et al., 2021;Gu et al., 2019;Yuan et al., 2013).

Yet the manner in which autonomic physiological fluctuations interact with fMRI measurements across gradual shifts in vigilance is not straightforward. Many studies treat vigilance and autonomic activity as separate influences on the fMRI signal (as reviewed inDuyn et al., 2020), but evidence suggests that autonomic fluctuations have stronger effects on fMRI signals when vigilance is tonically lower, for example, during rest versus task scans (Birn et al., 2006) and during rest with eyes closed versus open (Yuan et al., 2013). Vigilance-related changes in fMRI signals, including the marked increase in fMRI amplitude observed in drowsiness and the descent into sleep, may thus emerge at least in part from increased covariation between brain hemodynamics and autonomic fluctuations. Here, we investigate this possibility with a systematic analysis of simultaneous fMRI, EEG-determined vigilance levels, and autonomic recordings during both rest and a vigilance-probing auditory task. We assess how natural shifts in vigilance correspond to changes in fMRI signals and functional connectivity, and to the covariation between fMRI and autonomic signals. We hypothesize that covariation between autonomic physiological signals and fMRI fluctuations will increase as vigilance wanes, contributing to previously observed vigilance-related changes in fMRI amplitudes and inter-regional correlations.

## 2 Materials and Methods

### 2.1 Participants and data collection

This study was approved by the Institutional Review Boards of the National Institutes of Health and Vanderbilt University. Upon receiving written, informed consent, neuroimaging and autonomic data were collected from 14 healthy, right-handed adults (8 females, 6 males, mean ± S.D. age = 26.1 ± 4.4 years). Of these, nine participated in both rest and task scans (on the same day and in counterbalanced orders), two participated in only rest scans, and three participated in only the task. This resulted in 11 rest scans and 12 task scans.

Further details of the dataset are described inGoodale et al. (2021). Both the rest and task conditions lasted 24.5 min, using the same acquisition parameters. MRI data were acquired from a 3 T Siemens Prisma scanner (Siemens, Erlangen, Germany) with a Siemens 64-channel head/neck coil. For functional images, we used a multi-echo, gradient-echo EPI sequence with a flip angle of 75°; a repetition time (TR) of 2,100 ms; echo times (TEs) of 13.0, 29.4, and 45.7 ms; an in-plane acceleration factor of 2; a voxel size of 3 mm isotropic; a slice gap of 1 mm; a matrix size of 82 x 50; and 30 axial slices. For anatomical reference, we also collected a high-resolution, MP-RAGE T_1_-weighted structural image for each participant, using a flip angle of 9°, a TR of 2,200 ms, a TE of 4.25 ms, an inversion time of 1,000 ms, a voxel size of 1 mm isotropic, a matrix size of 256 x 256, and 160 sagittal slices.

During the fMRI scans, we acquired simultaneous scalp EEG data with a 32-channel MR-compatible system (BrainAmps MR, Brain Products GmbH) at a sampling rate of 5 kHz, synchronized to the MR scanner’s 10 MHz clock and using channel FCz as the reference. We also acquired simultaneous photoplethysmography (PPG) signals from the left index finger, and respiratory signals from a belt placed around the diaphragm, using MR-compatible transducers sampling at 2 kHz (Biopac, Goleta, CA). A separate transducer recorded MRI scanner triggers to enable data synchronization.

For the rest condition, participants’ only instructions were to keep their eyes closed and stay still and awake as best as possible. For the task condition, we used a psychomotor vigilance test (Lim & Dinges, 2008) described previously inGoodale et al. (2021). We delivered occasional, binaural tones via MR-compatible earbuds (VisuaStim Digital; Resonance Technology, Northridge, CA) with Presentation software (Neurobehavioral Systems, Berkeley, CA), and instructed participants to respond with a right-handed button press on an MR-compatible button box (Cambridge Research Systems, Rochester, UK) as soon as they heard a tone, in addition to keeping their eyes closed and staying as still and awake as possible. These tones occurred at long and randomized intervals to impede predictability and facilitate drifts in vigilance. For 5 of the 11 task participants, the tones occurred at inter-stimulus intervals of 29 to 41 s (mean ± S.D. = 34.74 ± 3.56 s); for the other 6, they occurred at inter-stimulus intervals of 8 to 89 s (mean ± S.D. = 42.45 ± 19.19 s). To ensure that the participants could reliably and comfortably hear the tones over the noise of the scanner, we calibrated the loudness level for each individual before beginning the task. Finally, we collected the stimulus and response data at a sampling rate of 100 Hz.

### 2.2 FMRI preprocessing

We discarded the first seven volumes (all echo times) of each scan to exclude any data collected before magnetization reached a steady state. We then performed slice-timing correction with the*3dTshift*function in the AFNI software suite (https://afni.nimh.nih.gov/afni), and motion correction with six-parameter rigid-body alignment, estimating the parameters from the middle echo time of each volume but applying them to all three with the function*3dvolreg*. Next, we used tedana 0.0.9a (DuPre et al., 2020) to clean the data of TE-independent signals (i.e., artifacts) via multi-echo independent components analysis (ICA). This procedure involved spatial ICA to decompose the multi-echo variance, sorting the resulting components according to whether or not their signals changed linearly across echo times, and then reconstructing the data with only those that demonstrated TE-dependence and are thus likely to reflect blood oxygen level-dependent (BOLD) changes with neuronal origins (seeKundu et al., 2012for more detail). This approach has proven highly effective at reducing fMRI noise from scanner drift, head motion, and non-BOLD physiological processes such as cardiac pulsatility and breath-to-breath respiration (Kundu et al., 2013), because these signals theoretically do not vary across echo times (Kundu et al., 2012). Note that it is unlikely to remove fMRI variance associated with low-frequency autonomic fluctuations, however, because they arise from T2* effects.

After multi-echo denoising, we transferred the fMRI volumes to the standard-space MNI152 template with non-linear registration using the*Normalise*module in SPM (https://www.fil.ion.ucl.ac.uk/spm/) and removed 0th- to 4th-order polynomial trends using AFNI’s*3dDetrend*function. For each participant, we then divided each voxel’s time series by its temporal mean value before detrending and multiplied the result by 100, arriving at units of percent signal change. Next, we spatially smoothed the data with a full-width half-maximum kernel of 3 mm using the*3dmerge*function in AFNI. Finally, because the imaging field of view excluded a small amount of brain matter from some participants, we masked out voxels missing data (0.85% of voxels from resting-state scans and 3.68% of voxels from task scans) from any participant to exclude these from our analyses.

### 2.3 Autonomic preprocessing

Following the procedures inChang et al. (2009),Özbay et al. (2019),Chen et al. (2020), andGoodale et al. (2021), we aligned the autonomic data to the timing of first fMRI trigger, and then calculated the respiratory volume (RV), heart rate (HR), and pulse wave amplitude (PWA) in sliding 6-s windows around each fMRI volume. For RV and PWA, we took the standard deviation of the respiratory and PPG time series, respectively, in each 6-s window, and then low-pass filtered these time series with a passband frequency of 0.15 Hz. For HR, we band-pass filtered the PPG data with a 2nd-order Butterworth filter from 0.5 to 2 Hz, detected peaks with a minimum height of 5% of the interquartile range and a minimum distance of 0.55 s (i.e., 109.09 beats per minute), and then calculated the time between each peak as the inter-beat interval (IBI). After visually inspecting the resulting IBI time series for artifacts (e.g., from poor peak detection) and interpolating over the few instances when these occurred (mean ± S.D = 1.12% ± 1.83% of IBIs per scan), we derived the HR measure as the inverse of the median IBI, converted to beats per minute, within each sliding window.

### 2.4 EEG preprocessing

We used BrainVision Analyzer 2 (Brain Products, Munich, Germany) for EEG preprocessing, following the procedures and parameters ofMoehlman et al. (2019)andGoodale et al. (2021). This involved reducing gradient artifacts by average artifact subtraction based on the fMRI triggers (Allen et al., 2000), downsampling the data to 250 Hz, and then reducing ballistocardiogram (BCG) artifacts by subtracting an artifact template locked to cardiac R-peaks (after accounting for the temporal difference between R-peaks and BCG effects) and using ICA on the resulting data to remove components likely reflecting BCG artifacts. We determined these components manually, based on their temporal deflections in relation to the cardiac cycle, their spatial topographies, and their contribution to the global field power, and constrained the number of noise components to no more than two per scan.

We extracted the average root-mean-squared amplitudes in the alpha (8–12 Hz), theta (3–7 Hz), and delta (0.5–2 Hz) frequency bands from the P3, Pz, P4, O1, Oz, and O2 channels during the acquisition time of each fMRI volume, averaging across channels to arrive at one value for each band at each TR. Following previous research (Goodale et al., 2021;Horovitz et al., 2008;Laufs et al., 2006), we then derived an electrophysiological vigilance index as the ratio of the power in the alpha versus theta bands.

### 2.5 Behavioral preprocessing

We removed the first 14.7 s (corresponding to the 7 discarded fMRI volumes) of the stimulus and response time series, and then measured the reaction time (RT) to each tone after this period as the time between each stimulus event and the following button press. If the response occurred after 4 s, we marked the RT as missing.

### 2.6 Behavioral validation of the EEG vigilance index

**Fig. 1.**
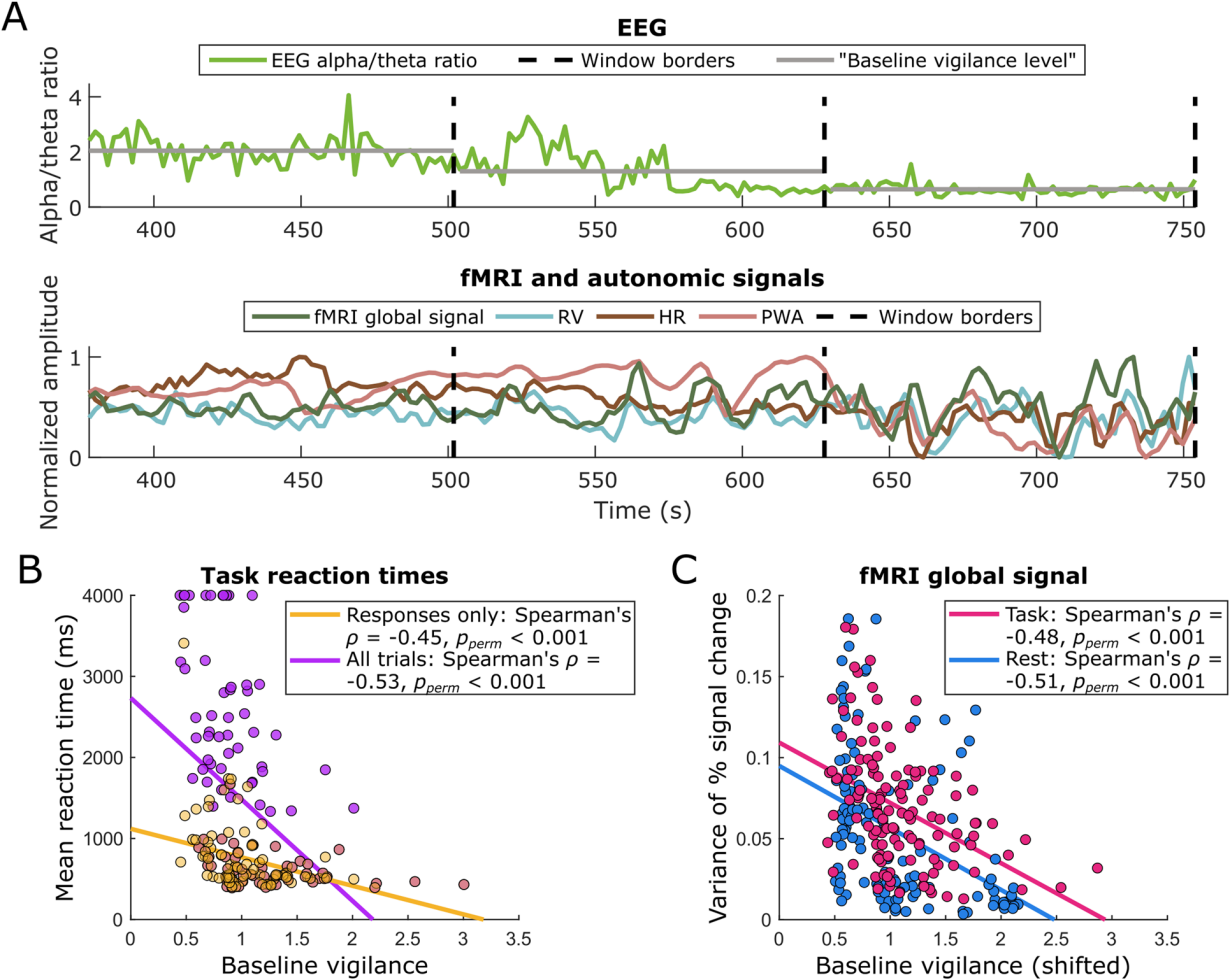
Comparing EEG, fMRI, autonomic, and behavioral measures across time windows. (A) Simultaneous EEG, fMRI, and autonomic data were divided into non-overlapping windows of 126 s each. This panel shows three representative, contiguous windows (the fourth, fifth, and sixth windows from rest participant 3), including their “fast” (i.e., seconds-level) and baseline (i.e., window-averaged) EEG alpha/theta ratios, for a participant in the process of falling asleep. (B) For the task scans, we compared the mean of the EEG alpha/theta power ratio within each window (which we define as “baseline vigilance”) to the mean reaction time in each window with Spearman’s rank correlations for non-normal distributions. Significant negative correlations, whether excluding trials without responses (“Responses only”) or including them as indicating arbitrarily long reaction times of 4 s (“All trials”), support the use of an EEG alpha/theta ratio as a measure of vigilance in this study. (C) The temporal variance of the percent signal change in the fMRI global signal also exhibited a negative relationship with baseline vigilance levels (shifted by 4.2 s in this case to accommodate the hemodynamic delay of the fMRI signal). This effect was significant for both resting-state and task data, indicating greater global fMRI variability as baseline vigilance decreases. Although the correlation values shown in (B–C) are based on non-parametric statistics, we include least-squares trend lines for visualization. RV = respiratory volume, HR = heart rate, PWA = pulse wave amplitude.

To measure gradual changes in vigilance, we divided the EEG alpha/theta time series into sliding, non-overlapping windows of 126 s, or 60 TRs (Fig. 1A), yielding 11 windows per participant. These windows contained 2–4 stimuli (mean ± S.D. = 3.15 ± 0.73) and 0–4 responses (mean ± S.D. = 2.48 ± 1.14) each (Supplementary Fig. S1). For each window from the task scans, we calculated the average RT to the stimuli that occurred within it and the average EEG vigilance index (i.e., “baseline vigilance”). Since neither of these variables was normally distributed, we compared them with Spearman’s rank correlations. In one version of this analysis, we treated missing RTs as missing data; in another, we replaced these values with 4 s (the nearest whole number above the longest RT of 3.41 s) to denote a maximal RT. Finally, we evaluated the two-tailed significance of these correlations with permutation tests based on shuffling the windowed vigilance values 10,000 times.

### 2.7 Comparing baseline vigilance to fMRI and autonomic variance

We again measured gradual changes in vigilance with sliding windows of 126 s each. For a global measure of fMRI activity, we measured the fMRI signal as the average of all voxels within the MNI152 template. We then divided the fMRI and autonomic signals into corresponding 126-s windows, after high-pass filtering with a passband frequency of 1/126 Hz to avoid inducing spurious fluctuations (Leonardi & van de Ville, 2015). We calculated the fMRI and HR variance within each window, excluding RV and PWA because their absolute values and therefore variance are arbitrary, varying according to the respiratory belt configuration, the force of the photoplethysmography sensor on the skin, etc. We then compared the fMRI and HR variances across windows to the baseline vigilance levels with Spearman’s rank correlations. We performed this analysis separately for the rest and task conditions and evaluated significance with 10,000 permutations. Since the fMRI BOLD signal is based on slow neurovascular coupling, we first shifted the EEG vigilance values by 4.2 s (2 TRs) to approximate the peak of ~5 s in canonical hemodynamic response functions, and then compared these to the windowed fMRI variance. Though the best-fitting lags between EEG and fMRI BOLD signals may differ by a few seconds (see, e.g.,de Munck et al., 2007), shifts of these magnitudes are negligible relative to our long sliding windows of 126 s each. Finally, we repeated this analysis while varying the window sizes from 30–120 TRs (i.e., 63–252 s) to assess its robustness across window sizes.

### 2.8 Evaluating fMRI-autonomic covariance across baseline vigilance levels

In each 126 s window, except for the two task windows with PWA outliers, we conducted a linear regression to assess the percentage of fMRI variance that could be explained by the autonomic measures. To do so, we convolved each participant’s RV time series with five basis functions to model the RV-to-fMRI transfer: a respiratory response function fromBirn et al. (2008), as well as two temporal derivatives and two dispersion derivatives as inChen et al. (2020). These steps preceded the removal of the first 14.7 s of data, to allow for lagged RV responses to bleed into the first sliding window. We similarly convolved the HR time series with a cardiac response function fromChang et al. (2009)along with two temporal derivatives and two dispersion derivatives as inChen et al. (2020), before discarding the first 14.7 s. For the PWA data, we convolved the time series with a canonical double-gamma hemodynamic response function as well as its temporal and dispersion derivatives using SPM’s*spm_get_bf*function, after shifting the data by 4.2 s (2 TRs) to eliminate the peak latency of the PWA-to-fMRI transfer. We then high-pass filtered the convolved autonomic and fMRI time series to avoid spurious fluctuations (Leonardi & van de Ville, 2015), and divided them into sliding windows of 126 s each. Within each one, we regressed the convolved autonomic regressors, standardized as*z*scores, against the fMRI time series to calculate the coefficient of determination (R^2^), that is, the proportion of fMRI variance explained. We then compared these values to the average EEG vigilance index in each window, shifted as above to accommodate the hemodynamic delay of the fMRI signal and its covariance, with Spearman’s rank correlation for non-parametric data. Finally, we tested the significance of this relationship with a two-tailed permutation test, shuffling the shifted baseline vigilance values 10,000 times to compute null correlations.

We performed this analysis separately for the task and rest conditions, and for the global fMRI signal (i.e., the average of all brain voxels) as well as multiple pre-defined networks of interest. These included the seven widely used cortical brain networks fromSchaefer et al. (2018), the subcortex (after removing any voxels that overlapped with the cortical atlas) as defined byTian et al. (2020), the white matter (based on the MNI152 template tissue priors, conservatively thresholded at 59.7% and limited to the cerebellum and cerebrum), and the ventricles (also based on the MNI152 template tissue priors, conservatively thresholded at 42.5%). We controlled for type I errors across these multiple comparisons with the Benjamini-Hochberg procedure, setting a false discovery rate (FDR) of 5%. For visualization, we calculated bootstrap 95% confidence intervals by randomly sampling from the sliding windows with replacement 2,000 times and then reevaluating the correlations between the sampled baseline vigilance and fMRI-autonomic covariance values. To evaluate the contribution of each autonomic measure individually, we also repeated this analysis with the global fMRI signal and the convolved response functions from one autonomic measure at a time.

To distinguish the fMRI and autonomic effects of vigilance changes from those elicited by the stimuli and button presses of the task condition, we included covariates for the stimulus events in the task linear regressions. We generated separate covariates for stimuli that did versus did not elicit responses, since these events are likely to elicit markedly different neural activity. This involved separating stimuli according to whether or not they were followed by a response, downsampling the binary (“on” or “off”) stimulus event time series from 100 Hz to 1 Hz, convolving them with the canonical double-gamma hemodynamic response function along with its temporal and dispersion derivatives with SPM’s*spm_get_bf*function, and then resampling the resulting time series to match the TR of 2.1 s before discarding the first 14.7 s as we had for the fMRI data. Since these functions account for a slow but temporally flexible hemodynamic lag of approximately 4 s, and the longest latency between a stimulus and a button press was 3.41 s, we did not include separate covariates for the button presses, which would have been highly collinear with the stimulus covariates. Instead, we included the convolved stimulus time series as regressors of no interest in the linear regressions for the task condition, after standardizing them as*z*scores. We then probed this analysis further by repeating it with window sizes varying from 30 to 120 TRs (i.e., 63–252 s) for the global fMRI signal and each network of interest.

We also tested for collinearity between the stimulus time series and the convolved autonomic measures, this time without distinguishing between stimuli that participants responded to or not. To do so, we regressed the autonomic measures against an all-stimuli time series that we had convolved with the canonical hemodynamic response function in each sliding window. These regressions explained a mean ± S.D. of 58.89% ± 12.18% of the variance in the stimulus regressor, indicating that including the stimuli in the regressions of fMRI-autonomic covariance would likely conceal much of the autonomic variance and thus the extent of fMRI-autonomic covariance. We therefore repeated the task analyses without the stimulus covariates, to compare its results with those from the rest condition and the task condition when modeling the stimulus events.

We followed these regional fMRI-autonomic covariance analyses with a voxelwise analysis to identify which specific areas of (or beyond) the networks of interest exhibited relationships with baseline vigilance. In this case, we increased the length of the sliding windows from 126 s to 241.5 s (115 TRs) and adjusted the high-pass filter passband frequency accordingly, to accommodate the noisiness of the fMRI signal in individual voxels. We also excluded voxels that were missing data for any participant.

We further probed these effects with supplementary analyses of their robustness. In the first case, we changed the size of the sliding windows for voxelwise analysis from 241.5 s to 126 s to match the size of the windows we used for global and network analyses. Another test involved calculating a different EEG vigilance metric, this time as the ratio of alpha/(delta + theta) power. We also reanalyzed these effects after constraining the sliding windows likely to contain sleep by excluding task windows in which participants failed to respond to the auditory stimuli and resting-state windows with baseline vigilance levels in the lowest tertile of all sliding windows. Finally, we constrained the sliding windows used for the task analysis to have baseline vigilance levels within the range of the baseline vigilance levels found during the resting-state scans.

### 2.9 Analyzing spatiotemporal dynamics of fMRI-autonomic covariance during high versus low baseline vigilance

We examined the spatiotemporal dynamics of the fMRI-autonomic relationships with cross-correlations using MATLAB’s x*cov*function with normalized scaling. This involved calculating the correlations between the high-pass filtered fMRI and each unconvolved autonomic signal, in each sliding window and from lags of -10.5 s (i.e., with the fMRI signal leading the autonomic signal by 5 TRs) to 31.5 s (i.e., with the autonomic signal leading the fMRI signal by 15 TRs). For the PWA analysis, we excluded the two windows with PWA outliers. Since this was a voxelwise analysis, we again used sliding windows of 241.5 s, and excluded voxels that were missing data for any participant. We then calculated the baseline vigilance index for each 241.5-s window in our sample, arriving at a whole-group distribution of baseline vigilance levels, and separated the distribution into thirds to identify those windows with the lowest (alpha/theta ratio ≤ 0.692), middle (0.692 < alpha/theta ratio < 1.0872), and highest (alpha/theta ratio ≥ 1.0872) baseline vigilance levels across participants. Finally, we computed the cross-correlations between the fMRI signals and each autonomic signal in each window, and then averaged the results for those windows in the bottom or top thirds of the pooled baseline vigilance levels.

The results of this analysis indicated dramatic differences between tissue types, and so we repeated this analysis after averaging the fMRI signals within the grey matter (Schaefer et al., 2018;Tian et al., 2020), white matter, or ventricle regions we had used to evaluate fMRI-autonomic covariance across baseline vigilance levels. We then calculated the mean and standard error of the cross-correlations in these networks. For completeness, we also evaluated cross-correlations between fMRI and EEG signals, sampled at the same rate of 1/2.1 Hz. Specifically, we examined the effects for the alpha/theta ratio and the alpha-, theta-, and delta-band powers.

### 2.10 Testing the role of more rapid electrophysiological fluctuations

To investigate the role of moment-to-moment fluctuations in electrophysiological power, we first examined cross-correlations between “fast,” seconds-level EEG alpha/theta ratios (sampled at each TR, or every 2.1 s) and each autonomic measure. To do so, we used MATLAB’s x*cov*function with normalized scaling on the time series of alpha/theta ratios (seeSection 2.4) and each autonomic time series in 126-s sliding windows, excluding the two windows with PWA outliers from the PWA cross-correlations. Analyzing lags of -10.5 s (i.e., with the fast EEG signal leading the autonomic signal by 5 TRs) to 31.5 s (i.e., with the autonomic signal leading the fast EEG signal by 15 TRs) and dividing the sliding windows into thirds as inSection 2.9, we then calculated the average cross-correlations during the windows in the lowest, highest, and middle thirds of the distribution of baseline (i.e., window-averaged) EEG alpha/theta ratios.

We then disentangled the effects of seconds-level EEG versus autonomic fluctuations on fMRI variance across baseline vigilance levels with a series of regressions. In one case, we formed seconds-level EEG regressors by convolving the time series of EEG power in the alpha, delta, and theta bands (sampled every 2.1 s, seeSection 2.4) with a basis set derived from the canonical double-gamma hemodynamic response function using SPM’s*spm_get_bf*function, and then discarded the first 14.7 s of the resulting regressors and high-pass filtered them for windowed analysis. We then assessed the relationship between baseline vigilance and fMRI-fast EEG covariance by calculating the proportion of fMRI variance explained by the seconds-level EEG regressors in each (shifted) sliding window (R^2^) and then correlating these R^2^values with the baseline vigilance levels using Spearman’s correlations and two-tailed permutation tests of 10,000 iterations. We repeated this analysis with autonomic variance partialled out of the fast EEG regressors, passing these partialled regressors to the analysis of fMRI-fast EEG covariance. Since we had nine seconds-level EEG regressors and 13 autonomic ones, we adjusted the R^2^values before comparison. We implemented these regression separately for grey matter (using the sum of all grey-matter cortical and subcortical regions of interest) (Schaefer et al., 2018;Tian et al., 2020), white matter, and the ventricles using the same definitions as inSection 2.8.

In a parallel analysis, we repeated this procedure with a basis set driven by the cross-correlations between seconds-level EEG and fMRI signals. Specifically, this basis set involved a double-gamma hemodynamic response function with a peak lag of 10 s, an undershoot lag of 14 s, peak and undershoot dispersion factors of 2, and a response-to-undershoot ratio of 2, modeled with SPM’s*spm_hrf*function (Supplementary Fig. S2). The set also included the temporal and dispersion derivatives of this basis function, with the first 14.7 s discarded and the resulting regressors high-pass filtered. As described above, we used these regressors to calculate the proportion of fMRI variance explained by the fast EEG regressors in each (shifted) sliding window (R^2^) and then correlated these proportions with the baseline vigilance levels using Spearman’s correlations and two-tailed permutation tests of 10,000 iterations. We again implemented this analysis separately for the grey matter, white matter, and the ventricles, and then repeated it with autonomic variance partialled out of the fast EEG regressors, adjusting the R^2^values for the number of regressors.

Finally, we evaluated regressions between fMRI-autonomic covariance and baseline vigilance levels with or without the fast EEG variance partialled out of the autonomic regressors. Again, we conducted this analysis in the grey matter, white matter, and ventricles separately, and adjusted the R^2^values for comparison.

### 2.11 Analyzing functional connectivity effects of baseline vigilance and autonomic variance

We assessed the functional connectivity (FC) between each pair of grey-matter networks (Schaefer et al., 2018;Tian et al., 2020) in sliding, 126-s windows by averaging and then high-pass filtering the fMRI time series in each network to avoid spurious fluctuations and then using Pearson’s correlations determine the relationships between these time series within each window. For the task condition, we computed these correlations with and without first regressing out the effects of the convolved stimulus covariates (seeSection 2.8). We then standardized the correlation coefficients with Fisher’s*z*transformation and used Spearman’s correlations for non-parametric data to relate these to the baseline vigilance index of each (shifted) window. We evaluated the significance of these correlations with two-tailed permutation tests of 10,000 iterations each and corrected for multiple comparisons with Benjamini-Hochberg’s procedure and a false discovery rate of 5%.

To explore the autonomic contribution to these results, we repeated the above analysis after regressing the convolved and*z*-scored autonomic signals out of the fMRI time series. We then compared the effects of gradual baseline vigilance shifts on FC in rest versus task, with and without this autonomic regression. This involved transforming the lower triangle of each 8 x 8 FC matrix to a 28-element vector, and then evaluating Pearson’s correlations between them with two-tailed permutation tests of 10,000 iterations.

## 3 Results

We assessed the relationships between simultaneously recorded fMRI, EEG, and peripheral autonomic data from 24.5-min sessions that spanned from relaxed wakefulness to light sleep (seeSection 2.1). We measured the EEG spectral power, respiratory volume (RV), heart rate (HR), and pulse wave amplitude (PWA) at each BOLD fMRI time point across the scan (TR = 2.1 s; seeSections 2.1,2.3, and2.4). The dataset included resting-state scans from 11 participants, and for 12 participants, vigilance was probed with intermittent auditory tones. Participants were instructed to keep their eyes closed and respond to tones with a right-handed button press as soon as possible. The tones were presented at long and highly variable inter-stimulus intervals to render them unpredictable and facilitate fluctuations in vigilance over time (seeSection 2.1).

### 3.1 FMRI, peripheral autonomic signals, and reaction times change across baseline vigilance levels

To investigate whether different vigilance levels are associated with differences in the characteristics of fMRI and peripheral autonomic signals, we divided the fMRI, EEG, and autonomic data (plus the behavioral data for the task participants) into sliding, non-overlapping windows of 126 s each (seeFig. 1A). This resulted in 11 windows per participant. We indexed the vigilance level for each window as the mean of the EEG alpha/theta power ratio within it (Bodala et al., 2016;Cote et al., 2009;Goodale et al., 2021;Horovitz et al., 2008;Laufs et al., 2006), which we hereafter refer to as “baseline vigilance.” To evaluate any changes in fMRI, autonomic, or behavioral responses across systematically varying levels of baseline vigilance, we then conducted Spearman’s rank correlations for non-normal distributions and assessed significance with permutation tests. We observed that windows with higher baseline vigilance levels tended to have shorter average task reaction times. This was true regardless of whether we excluded missing responses from the analysis (Spearman’s*ρ*= -0.45,*p_perm_*< 0.001;Fig. 1B, yellow dots) or encoded them as arbitrarily late responses of 4 s (Spearman’s*ρ*= -0.53,*p_perm_*< 0.001;Fig. 1B, purple dots), supporting the use of a window-averaged EEG alpha/theta ratio as a proxy measure for baseline vigilance.

Across sliding windows, we then compared the baseline vigilance index to the variability of the global fMRI signal (i.e., the temporal variance of the whole-brain averaged percent signal change time course in each sliding window; seeSection 2.2). Since fMRI hemodynamic responses lag several seconds behind electrophysiological activity, we calculated the baseline vigilance levels for this analysis after shifting the EEG power signals forward in time by 2 TRs (4.2 s) to approximate the lag of a peak hemodynamic response. Lower baseline vigilance was associated with significantly higher global fMRI variance for both the task and rest conditions (task Spearman’s*ρ*= -0.48,*p_perm_*< 0.001; rest Spearman’s*ρ*= -0.51,*p_perm_*< 0.001;Fig. 1C). Comparing baseline vigilance levels to autonomic variance at 0 lag (i.e., instantaneously), we found that lower baseline vigilance was associated with more variable HR during the task (Spearman’s*ρ*= -0.27,*p_perm_*= 0.002) but less variable HR during rest (Spearman’s*ρ*= 0.19,*p_perm_*= 0.035). We excluded RV and PWA variance from this analysis due to the arbitrary values of the respiratory belt and photoplethysmography signals (seeSection 2.7).

### 3.2 The association between fMRI and autonomic signals increases as baseline vigilance diminishes

**Fig. 2.**
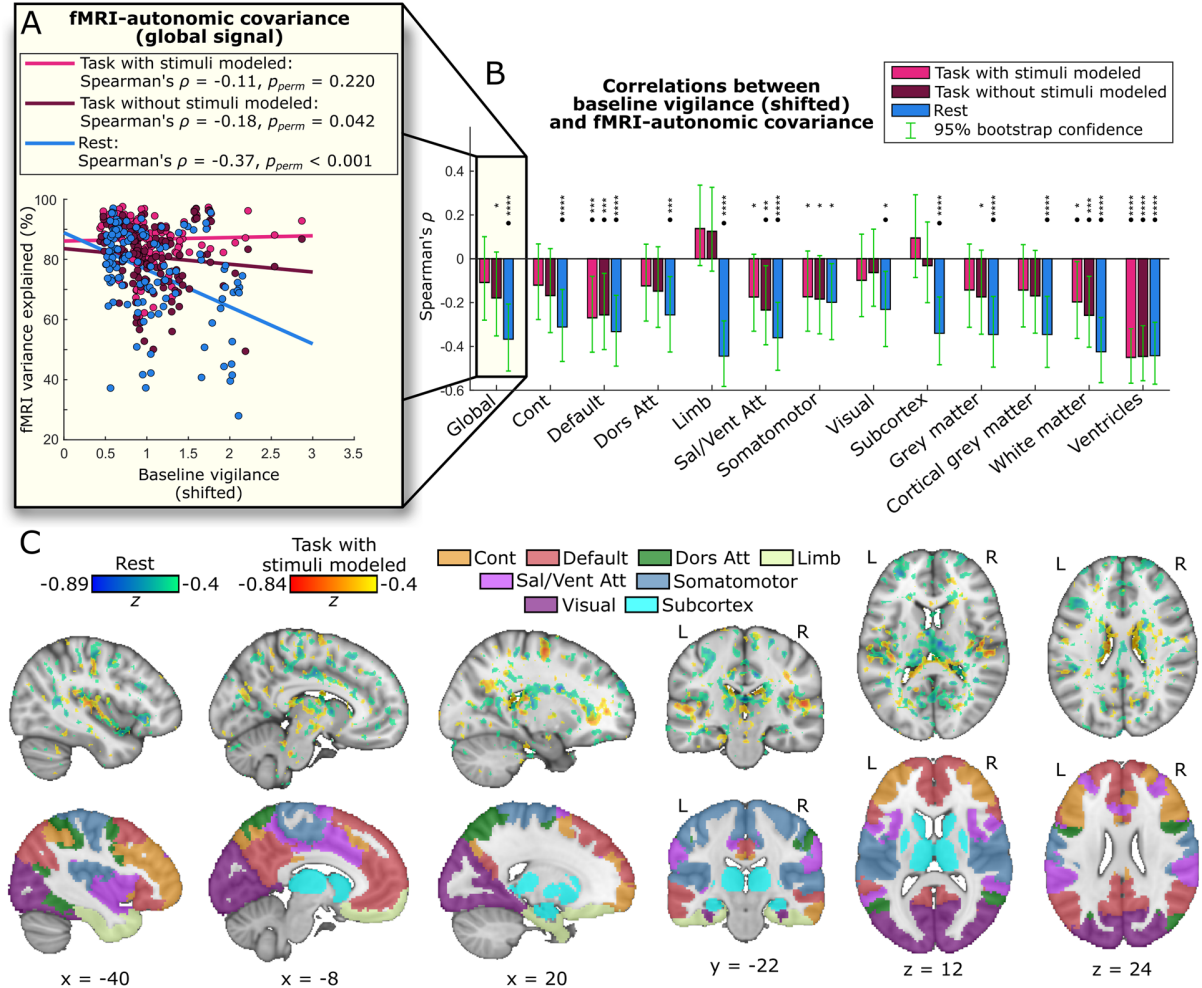
Lower baseline vigilance is associated with greater fMRI-autonomic covariance. (A) In non-overlapping, sliding windows, we performed linear regressions to evaluate the percentage of fMRI variance that could be explained by autonomic signals. We then correlated these values with the mean (i.e., baseline) vigilance level for each window, shifting the windows by 4.2 s (2 TRs) to accommodate the hemodynamic lag of the fMRI signal. For the task data, we performed this analysis with (“Task with stimuli modeled”) and without regressors for the stimulus events (“Task without stimuli modeled”). Although these correlation values are based on non-parametric statistics, we include least-squares trend lines for visualization. (B) We conducted this analysis for the global fMRI signal and for several predefined brain networks in cortical (Schaefer et al., 2018) and subcortical (Tian et al., 2020) grey matter, white matter, and ventricles. Correcting for multiple comparisons with a 5% false discovery rate, we identified several networks with significantly greater fMRI-autonomic covariance during lower baseline vigilance levels, especially in the resting-state condition. (C) We also examined this effect in each voxel, revealing the many areas with greater autonomic contributions during lower baseline vigilance throughout resting state and the psychomotor vigilance task (shown here only with stimuli modeled for simplicity). The top row of this panel depicts voxelwise effects, and the bottom row shows the networks of interest overlaid on the same brain slices. Global = global signal, Cont = control network, Default = default mode network, Dors Att = dorsal attention network, Limb = limbic network, Sal/Vent Att = salience/ventral attention network, Somatomotor = somatomotor network, Visual = visual network, * =*p_perm_*≤ 0.05, ** =*p_perm_*≤ 0.01, *** =*p_perm_*≤ 0.005, **** =*p_perm_*≤ 0.001, • = survives multiple-comparisons correction with a false discovery rate of 5%.

We then assessed whether vigilance also modulates the strength of the relationship between fMRI and autonomic signals. To do so, we convolved the autonomic signals with basis sets generated from previously derived hemodynamic response functions (seeSection 2.8) (Birn et al., 2008;Chang et al., 2009;Chen et al., 2020) and then regressed the resulting waveforms (“autonomic regressors”) against the fMRI activity in each sliding window. This procedure resulted in coefficients of determination (R^2^s), signifying the proportion of fMRI variance that could be explained by the autonomic regressors—that is, the fMRI-autonomic covariance—in each time window. We converted these values to percentages and then compared them to the baseline vigilance level of each window, shifted forward by 4.2 s (2 TRs) to accommodate for hemodynamic delay (seeFig. 2A). We conducted this analysis with the global fMRI signal as well as several predefined regions of interest: seven canonical brain networks (Schaefer et al., 2018), the subcortex (Tian et al., 2020), grey matter (GM), cortical GM only, white matter (WM), and the third and lateral ventricles (Fig. 2B).

Since the task included auditory stimuli with button-press responses, some of the changes in fMRI signals during the task may be driven by these events. We therefore included regressors for the stimuli in the analyses of fMRI-autonomic covariance, separately modeling stimuli that did or did not elicit responses, to remove the fMRI variance associated with these task events (further details inSection 2.8). In so doing, we observed that the time series of stimulus events convolved with the canonical hemodynamic response function were highly collinear with the autonomic regressors, implying that the stimuli elicited autonomic responses: indeed, the autonomic regressors explained a mean ± S.D. of 58.89% ± 12.18% of the variance in convolved time series of all stimuli. Between the individual autonomic measures, RV exhibited the closest association with stimulus time courses (29.22% ± 14.78% variance explained), followed by HR (23.36% ± 12.99%) and PWA (14.63% ± 10.56%). Including stimulus regressors when assessing fMRI-autonomic covariance would therefore remove a substantial fraction of autonomic variance, and so we conducted parallel analyses without the stimulus regressors to leave the autonomic signals intact. We include the results of both analyses for side-by-side comparison (Fig. 2A, B).

Altogether, these analyses reveal increased autonomic contributions to fMRI signals in widespread areas of the brain during lower levels of baseline vigilance (Fig. 2). Correcting for multiple comparisons in the predefined networks of interest with a false discovery rate (FDR) of 5%, we identified significant effects in the resting-state condition in the global signal (Spearman’s*ρ*= -0.37,*p_perm_*< 0.001), control network (Spearman’s*ρ*= -0.31,*p_perm_*< 0.001), default mode network (Spearman’s*ρ*= -0.33,*p_perm_*< 0.001), dorsal attention network (Spearman’s*ρ*= -0.25,*p_perm_*= 0.006), limbic network (Spearman’s*ρ*= -0.44,*p_perm_*< 0.001), salience/ventral attention network (Spearman’s*ρ*= -0.36,*p_perm_*< 0.001), visual network (Spearman’s*ρ*= -0.22,*p_perm_*= 0.014), subcortex (Spearman’s*ρ*= -0.33,*p_perm_*< 0.001), entire GM (Spearman’s*ρ*= -0.34,*p_perm_*< 0.001), cortical GM (Spearman’s*ρ*= -0.34,*p_perm_*< 0.001), WM (Spearman’s*ρ*= -0.42,*p_perm_*< 0.001), and ventricles (Spearman’s*ρ*= -0.44,*p_perm_*< 0.001). Significant effects in the task condition were found in the default mode network (Spearman’s*ρ*= -0.26,*p_perm_*= 0.003), salience/ventral attention network (Spearman’s*ρ*= -0.23,*p_perm_*= 0.008), WM (Spearman’s*ρ*= -0.26,*p_perm_*= 0.004), and ventricles (Spearman’s*ρ*= -0.45,*p_perm_*< 0.001) when excluding stimulus covariates, and to the default mode network (Spearman’s*ρ*= -0.27,*p_perm_*= 0.002), WM (Spearman’s*ρ*= -0.20,*p_perm_*= 0.021), and ventricles (Spearman’s*ρ*= -0.45,*p_perm_*< 0.001) when including them. These vigilance-associated effects were robust across a range of different sliding-window sizes, indicating that the choice of 126 s could not explain these results (Supplementary Fig. S3).

While the primary statistical comparison was carried out at the region-wise level (Fig. 2A), we also examined a more spatially resolved view of the corresponding effects by performing this analysis in each brain voxel separately. To mitigate the effects of increased noise associated with extracting signals from individual voxels, we used longer but still non-overlapping windows of 241.5 s (115 TRs). Since the network-level effects for the task condition without stimuli modeled were almost uniformly between those for the task with stimuli modeled and the resting-state condition, we used only the task with stimuli modeled and resting-state conditions for this analysis (Fig. 2C). We assessed the robustness of these results by replicating this analysis in windows of 126 s each (to match those used for global- and networked-based analyses), and found comparable effects in the resting-state data along with weaker but topographically similar effects in the task data (Supplementary Fig. S4). Furthermore, we tested whether a different EEG vigilance metric would change these results. Given the association between the power of the delta frequency band and vigilance (T. T. Liu & Falahpour, 2020;Merica & Fortune, 2004;Ogilvie, 2001;Soon et al., 2021), we used the ratio of alpha power to power in the combined delta-to-theta range to derive a different EEG vigilance index (seeSection 2.8), and found the relationships between baseline vigilance calculated with this index and fMRI-autonomic covariance largely unchanged (Supplementary Fig. S5).

To examine the individual role of each autonomic measure in the observed relationship between baseline vigilance and fMRI-autonomic covariance, we repeated this analysis for RV, HR, and PWA separately (Table 1). In this case, we examined only the global fMRI signal for simplicity. While the covariance between fMRI and RV signals was not significantly related to baseline vigilance for either the rest or task (with or without stimuli modeled) conditions (*p*s ≥ 0.094), fMRI-HR covariance significantly increased with decreasing baseline vigilance during both rest (Spearman’s*ρ*= -0.45,*p_perm_*< 0.001) and the task (with stimuli-modeled Spearman’s*ρ*= -0.25,*p_perm_*= 0.005; without stimuli-modeled Spearman’s*ρ*= -0.33,*p_perm_*< 0.001). FMRI-PWA covariance also significantly increased as baseline vigilance decreased during both rest (Spearman’s*ρ*= -0.38,*p_perm_*< 0.001) and the task without stimuli modeled (Spearman’s*ρ*= -0.18,*p_perm_*= 0.036), but this effect was not significant in the task condition when including stimulus regressors (*p_perm_*= 0.097).

**Table 1.** Relationship between baseline vigilance and global signal fMRI-autonomic covariance.

Measurement	Condition	Spearman's *ρ*	* p _perm_*
Respiratory volume (RV)	Task with stimuli modeled	-0.05	0.577
	Task without stimuli modeled	-0.12	0.156
	Rest	-0.15	0.094
Heart rate (HR)	Task with stimuli modeled	-0.25	0.005 •
	Task without stimuli modeled	-0.33	<0.001 •
	Rest	-0.45	<0.001 •
Pulse wave amplitude (PWA)	Task with stimuli modeled	-0.15	0.097
	Task without stimuli modeled	-0.18	0.036
	Rest	-0.38	<0.001 •

• = survives multiple-comparisons correction with a false discovery rate of 5%.

Finally, to ensure that the above vigilance-related associations are not likely due to differences in head motion across vigilance levels, we calculated the mean framewise displacement (FD) of head motion within each temporal window and correlated this across sliding windows with the mean EEG vigilance index, the mean variance in the global fMRI signal or autonomic signals, and the fMRI-autonomic covariance. All uncorrected*p*values were non-significant (*p = *0.059 for the relationship between mean FD and the global fMRI signal variance, and*p*s ≥ 0.138 for all other comparisons).

### 3.3 Spatiotemporal dynamics of fMRI-autonomic covariance during low and high baseline vigilance

**Fig. 3.**
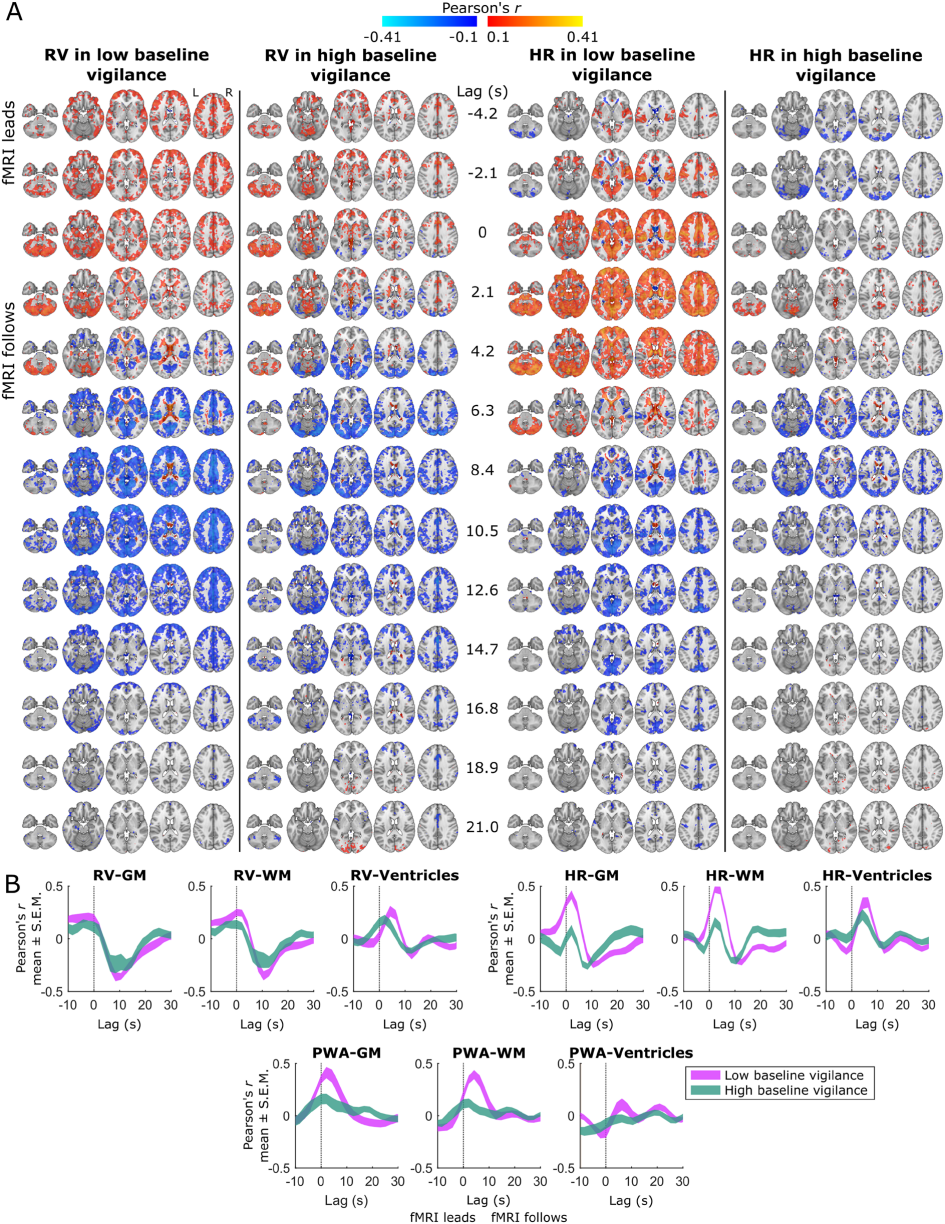
Spatiotemporal dynamics of fMRI-autonomic correlations during low and high baseline vigilance across voxels and tissue types. (A) To identify where and when fMRI-autonomic correlations occur in low versus high baseline vigilance, we calculated the Pearson cross-correlation between the fMRI signal in each voxel and each autonomic signal during the sliding windows with the lowest and highest baseline vigilance levels, at lags from -10.5 to 31.5 s. The axial slices shown here are at MNI z values -36, -18, 0, 18, and 36, with the left hemispheres on the left. Maps are averaged over subjects. (B) We then averaged the voxelwise values separately for grey matter (GM), white matter (WM), and third/lateral ventricles (Ventricles), and compared the effects in low versus high baseline vigilance. Curves illustrating the range of mean ± the standard error of the mean (S.E.M.) for each cross-correlation show that these effects were generally larger during low baseline vigilance, especially for heart rate (HR) and pulse wave amplitude (PWA) in the GM and WM and less so for respiratory volume (RV).

We examined changes in fMRI-autonomic covariance across baseline vigilance levels in greater spatiotemporal detail by analyzing cross-correlations between fMRI signals and each autonomic measure. Focusing on the resting-state scans because they showed the strongest vigilance-related modulation of fMRI-autonomic covariance, and using 241.5-s (115-TR) sliding windows again for voxelwise analysis, we compared the effects that occurred in the windows with the lowest versus highest baseline vigilance levels. Specifically, we identified windows whose baseline vigilance levels fell within the top or bottom third of all observed values (pooled across participants). We then averaged the fMRI-autonomic cross-correlations across the windows with the lowest baseline vigilance levels and did the same across the windows with the highest baseline levels. This approach illustrates the spatial distribution of fMRI signals correlated with each autonomic variable at these contrasting baseline vigilance levels, without the assumption of a specific hemodynamic response shape (Fig. 3A).

Increasing RV was associated with moderate fMRI signal increases starting before or at lag 0 (instantaneously). Averaging the cross-correlations for each tissue type revealed that the RV-fMRI correlation in the GM became negative after about 2 to 4 s, while reaching positive peaks in the WM and ventricles at these latencies. Ultimately, all fMRI-RV correlations exhibited negative peaks after about 8.4 to 12.6 s. This pattern was similar for the low- and high-vigilance windows, though with slightly stronger correlations during lower baseline vigilance (Fig. 3B). HR and PWA exhibited similar effects, with changes in either of these signals corresponding to positively correlated and relatively strong fMRI changes in GM and WM, but negligible or negatively correlated changes in the ventricles at lag 0. These correlations all became more positive over the next few seconds, such that there were widespread positive correlations throughout the GM and WM from about 2.1 to 6.3 s, which dropped off just as positive ventricle correlations emerged at about 4.2 to 8.4 s. As with RV, these effects were stronger during low versus high baseline vigilance (Fig. 3B). For completeness, we also evaluated cross-correlations between fMRI and the EEG power signals sampled at each TR (seeSection 2.9) and present these results inSupplementary Figure S6.

### 3.4 Contributions of more rapid electrophysiological fluctuations

The results discussed so far support the hypothesis that increased fMRI signal variability during periods of lower vigilance may arise to some extent from vigilance-linked changes in autonomic activity. In these analyses, baseline vigilance levels were obtained by averaging EEG alpha/theta ratios across minutes-long windows and comparing faster (seconds-level) fluctuations in fMRI and autonomic signals within these windows. Yet neuronal activity and electrophysiological markers thereof—such as alpha-, theta-, and delta-band power—also exhibit prominent fluctuations on these more rapid timescales (seeFig. 1A). In fact, brief neurophysiological events like microsleeps and k-complexes occur more frequently as wakefulness declines (Hertig-Godeschalk et al., 2020;Merica & Fortune, 2004;Ogilvie, 2001;Soon et al., 2021;Wong et al., 2013), and these are notably accompanied by changes in autonomic physiology and a spatiotemporal pattern of fMRI signals that closely resemble the effects of autonomic fluctuations (X. Liu et al., 2018;Özbay et al., 2019;Picchioni et al., 2022;Soon et al., 2021;Yuan et al., 2013). Such “fast” (i.e., seconds-level) neural fluctuations are thus likely to account for at least some portion of fMRI variance (Gu et al., 2022), and may contribute to the observed increases in fMRI signal variability during lower baseline vigilance levels. We therefore examined EEG power fluctuations sampled at the TR of 1/2.1 Hz autonomic and fMRI signals as baseline vigilance decreased.

**Fig. 4.**
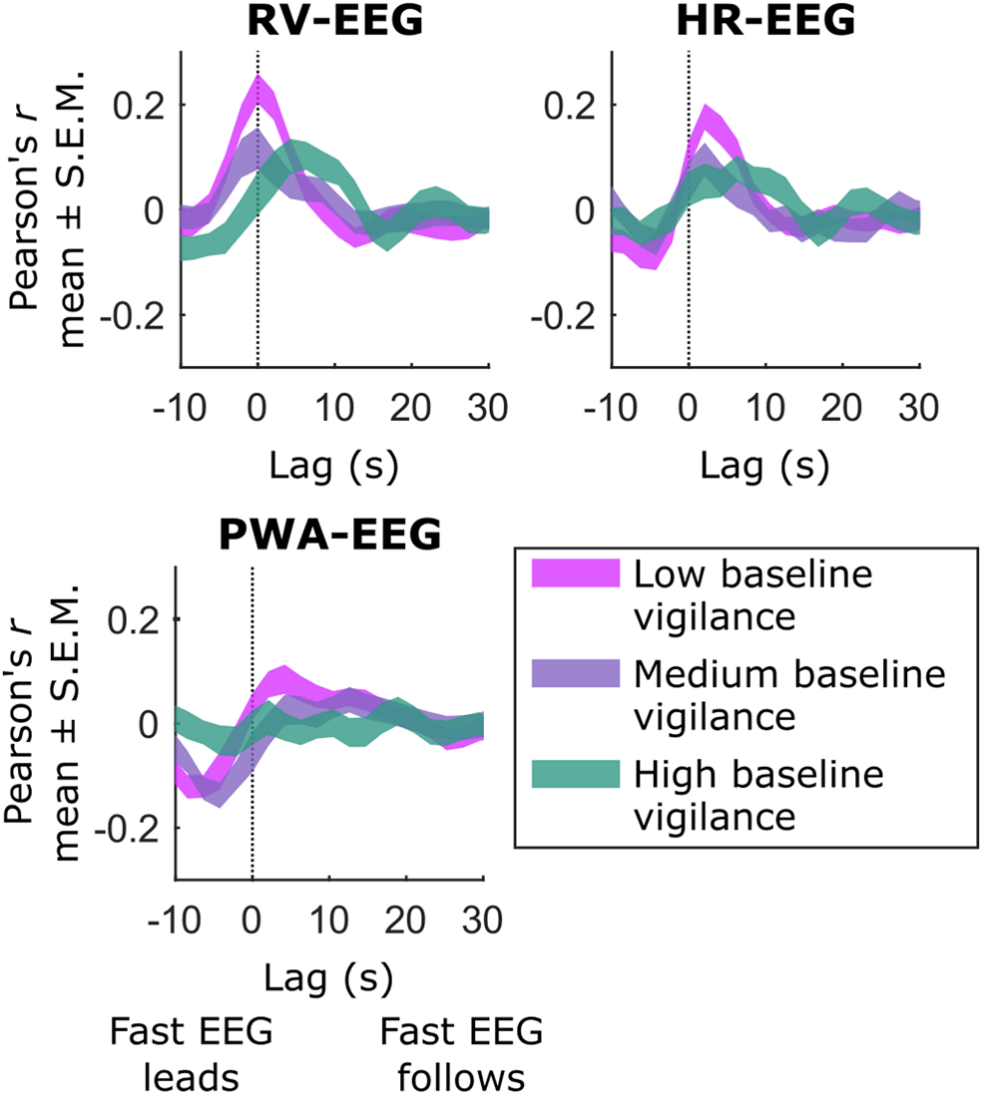
Correlations between fast (seconds-level) EEG power (alpha/theta ratio) and autonomic measures vary over time and baseline (minutes-level) EEG vigilance levels. We assessed the relationship between seconds-level EEG power (“fast EEG”) measures and autonomic signals with Pearson’s correlations at lags from -10.5 to 31.5 s, and averaged these correlations separately for the sliding windows in the lowest (“low baseline vigilance”), highest (“high baseline vigilance”), and middle thirds of the distribution of observed baseline vigilance levels. Curves illustrating the range of mean ± the standard error of the mean (S.E.M.) for each cross-correlation revealed moderate correlations between fast EEG fluctuations on the one hand and respiratory volume (RV), heart rate (HR), or pulse wave amplitude (PWA) on the other, which became stronger as baseline vigilance decreased.

First, we evaluated whether “fast,” seconds-level EEG power and peripheral autonomic fluctuations become more strongly correlated to one another as baseline, minutes-level EEG measures of vigilance decrease. Using only the resting-state data, we computed cross-correlations between the fast EEG power signals and each autonomic signal within each 126-s sliding window. We then categorized the windows according to their baseline vigilance levels (i.e., average EEG alpha/theta ratios), identifying those with the lowest, middle, and highest values (seeSection 2.9). Averaging the cross-correlations for each of these three baseline vigilance conditions indicated that moderate relationships between fast EEG changes and autonomic measures did, in fact, strengthen when baseline vigilance was lower (Fig. 4).

We then explored how the relationship between fast EEG power fluctuations and fMRI signals varies across different baseline vigilance levels. Using regressions similar to those described above and portrayed inFigure 2A, we calculated the proportion of fMRI variance (R^2^) that could be explained by regressors of fast EEG signals in each sliding window, formed by convolving alpha-, theta-, and delta-power time series (again sampled every 2.1 s) with basis sets to model their hemodynamic responses. We approached this modeling in two ways: first using a standard, canonical hemodynamic response function and second with an empirically derived response function based on the cross-correlations between fast EEG alpha/theta power and fMRI signals (Supplementary Fig. S6). The latter is motivated by prior research that reported longer delays between EEG power and fMRI signals in a number of brain areas (de Munck et al., 2007) (seeSection 2.10;Supplementary Fig. S2). With each of these approaches, we explored the portion of fast EEG fluctuations that was orthogonal to autonomic fluctuations by partialling autonomic regressors out of the fast EEG regressors. We also reexamined fMRI-autonomic covariance after partialling out fast EEG regressors, to discriminate the unique effects of autonomic signals from those of fast EEG fluctuations.

**Fig. 5.**
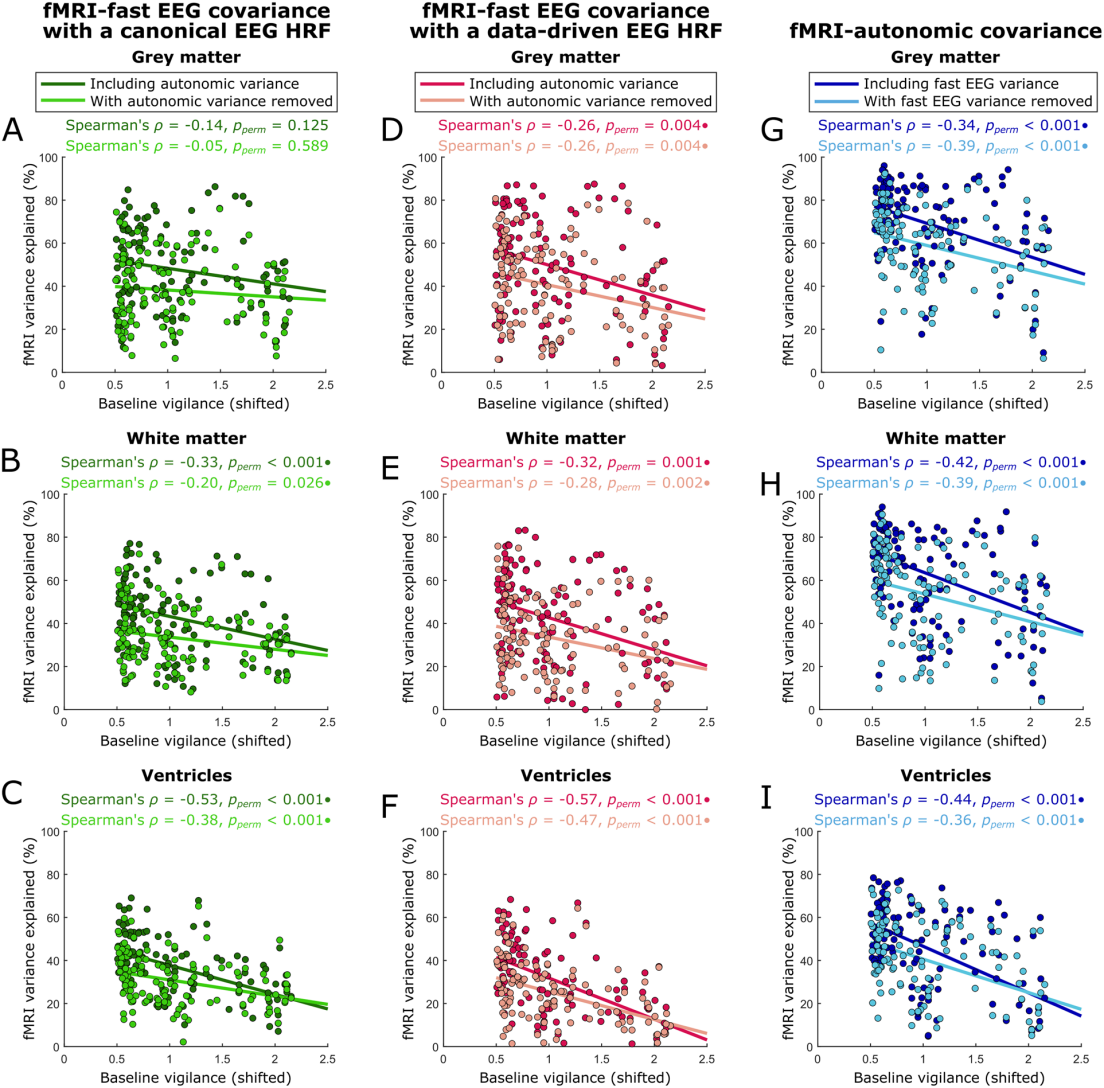
Relationships between fast (seconds-level) EEG power fluctuations and fMRI signals across baseline vigilance levels, in comparison to autonomic effects. We measured the amount of fMRI variance explained by fast (seconds-level) EEG and autonomic regressors in sliding windows across baseline (minutes-long) vigilance levels, and then distinguished between these effects with orthogonalization. (A–C) Convolving time series of fast EEG power in the alpha, delta, and theta bands (sampled every 2.1 s) with a basis set derived from the canonical hemodynamic response function (HRF), we found strong correlations between baseline vigilance and fMRI-fast EEG covariance in the white matter and ventricles but not the grey matter (dark green). These effects were moderately weakened by partialling autonomic variance out of the fast EEG regressors (light green). (D–F) Similarly, convolving fast EEG time series with a “data-driven” HRF basis set derived from the cross-correlations between fMRI and fast EEG signals (Supplementary Fig. S4) indicated strong correlations between baseline vigilance and fMRI-fast EEG covariance in all three tissue types (red), which only slightly lessened after partialling out autonomic variance (salmon). (G–I) Correlations between baseline vigilance and fMRI-autonomic covariance were also significant in the grey matter, white matter, and ventricles (dark blue), even when the autonomic signals were orthogonalized with respect to fast EEG fluctuations (light blue, here, using the data-driven EEG basis set). Although these correlation values are based on non-parametric statistics, we include least-squares trend lines for visualization. • = survives multiple-comparisons correction with a false discovery rate of 5%.

These analyses revealed greater covariation between fMRI and fast EEG signals as baseline vigilance decreased (Fig. 5A–F, dark data). This effect was strongest in the ventricles and with the data-driven EEG response function (i.e., that based on the fMRI-EEG cross-correlations), and failed to reach significance in the grey matter when convolving EEG power with the canonical HRF. Partialling out autonomic variance slightly weakened this effect (Fig. 5A–F, light data), especially when the EEG convolution was based on the canonical hemodynamic response function. Similarly, the previously described and strong relationships between baseline vigilance and fMRI-autonomic covariance were only marginally affected by the removal of fast EEG variance (Fig. 5G–I). Though idiosyncrasies of the different measurements (such as their respective signal-to-noise ratios) may color these effects (seeSection 4.2), these results suggest that changes in fMRI signals across baseline vigilance levels are associated with both fast electrophysiological and autonomic fluctuations, sharing a unique portion of variance with each.

### 3.5 Impact of vigilance-related autonomic effects on fMRI functional connectivity

**Fig. 6.**
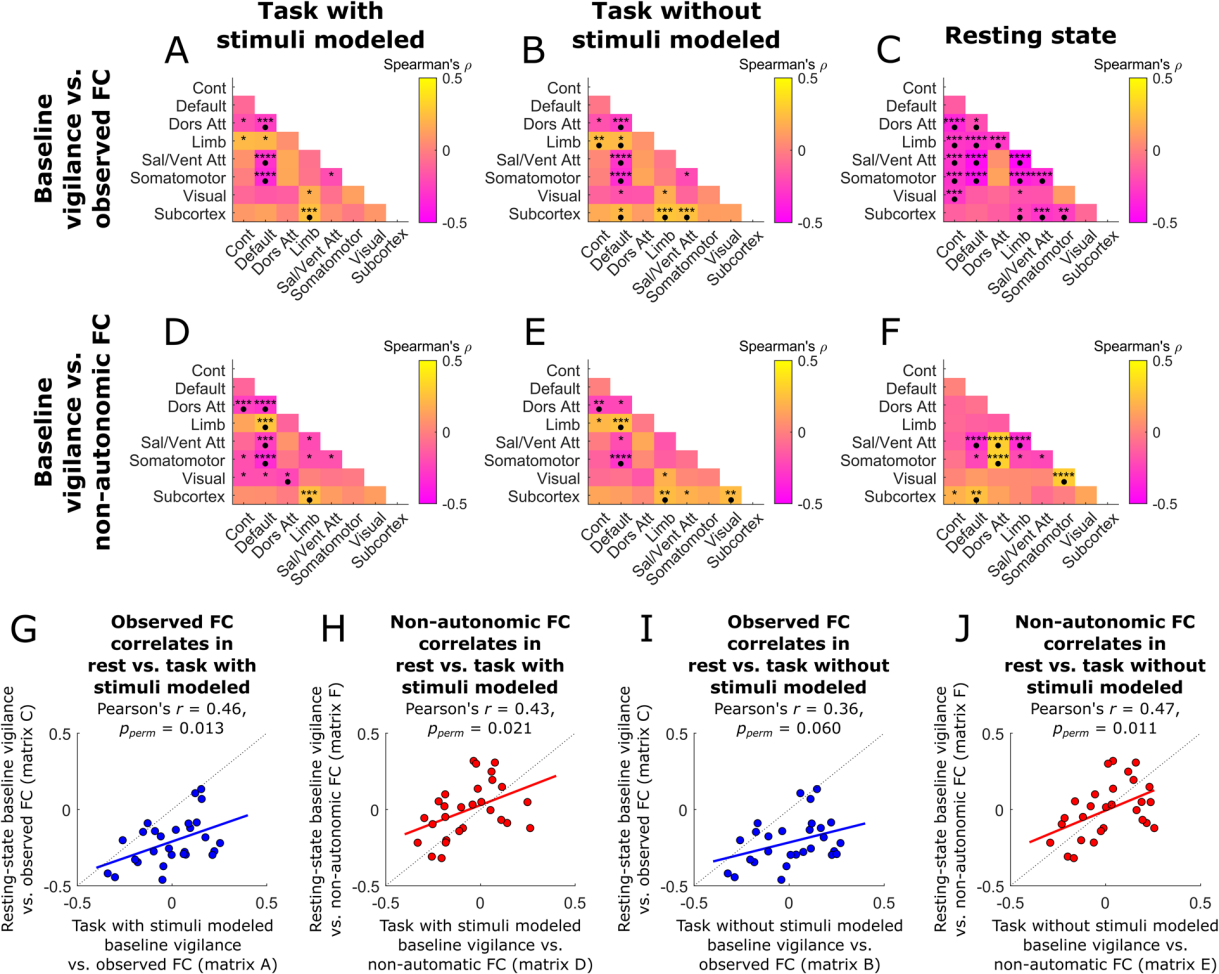
Baseline vigilance is associated with functional connectivity changes throughout the brain, much of which shares variance with autonomic effects. For resting state and the vigilance task (with and without stimuli modeled), we evaluated the functional connectivity (FC) associated with baseline vigilance (A–C) and then repeated this analysis after regressing out autonomic variance (D–F). This analysis revealed significant changes in FC as baseline vigilance waned, especially for the default mode network. Further, removing autonomic signals had a greater impact on vigilance-linked FC in the resting-state versus task condition. We then directly assessed the similarity between vigilance-linked FC changes during the resting-state versus task FC conditions, and examined how this correspondence was altered after partialling out stimulus and/or autonomic signals (G–J). FC patterns were largely similar across conditions whether or not we modeled the task stimuli, but became more similar when we removed the effects of autonomic variance. These results suggest that the differences between task and resting-state effects are at least partially explained by differences in autonomic activity. Cont = control network, Default = default mode network, Dors Att = dorsal attention network, Limb = limbic network, Visual = visual network, * =*p_perm_*≤ 0.05, ** =*p_perm_*≤ 0.01, *** =*p_perm_*≤ 0.005, **** =*p_perm_*≤ 0.001, • = survives multiple-comparisons correction with a false discovery rate of 5%.

Vigilance- and autonomic-related changes in fMRI signals can have profound effects on the correlations (functional connectivity; FC) between brain regions (Chen et al., 2020;Wang et al., 2016;Xifra-Porxas et al., 2021). We therefore first examined FC during each sliding window, using the grey-matter networks defined above (Schaefer et al., 2018;Tian et al., 2020), and then compared these values to their respective baseline vigilance levels (seeSection 2.11). In the task condition, baseline vigilance was negatively correlated with FC between the default mode, salience/ventral attention, and somatomotor networks, and between the default mode and dorsal attention networks, and was positively correlated with FC from the limbic network and subcortex to other networks. These effects were largely consistent whether stimulus regressors were included (Fig. 6A) or not (Fig. 6B). In the rest condition, however, baseline vigilance was negatively associated with FC between most network pairs (Fig. 6C). We compared these patterns of vigilance-related FC between the task and rest conditions by reshaping each FC matrix into a vector and then correlating the results for task versus rest data. This analysis revealed similar FC patterns between rest and the task with stimuli modeled (Fig. 6G), but less similar FC patterns during rest and the task without stimuli modeled (Fig. 6I). In each of these comparisons, the effects during resting state were almost universally more negative.

To gauge the extent to which autonomic variance contributes to the effect of baseline vigilance on FC, we repeated the above analysis but this time after partialling out the (convolved) autonomic variance from the fMRI time series. Doing so left the relationship between baseline vigilance and FC relatively unchanged in the task condition (compareFig. 6Dvs.Fig. 6AandFig. 6Evs.Fig. 6B) but limited the scope of significant baseline vigilance effects on FC in the resting-state condition from 16 to 6 network pairs, most of which included the salience/ventral attention, dorsal attention, and/or somatomotor networks (compareFig. 6Fvs.Fig. 6C). Partialling out autonomic variance had a negligible effect on the similarity of FC patterns between rest and the task with stimuli modeled (Fig. 6H) but made the FC patterns between the resting state and the task without stimuli modeled more alike (Fig. 6J). Given the high (~60%) collinearity between the stimulus covariates and the autonomic signals (seeSection 3.2), these results imply that much of the difference in vigilance-associated FC changes between the rest and task conditions may be explained by autonomic differences, which are obscured by the stimulus regressors when those are included in the analysis.

## 4 Discussion

Disentangling the determinants of cerebral blood oxygen levels is essential for understanding brain function, and for interpreting hemodynamic imaging measurements such as fMRI. Here, we conduct a systematic and integrated analysis of two major, interrelated influences on fMRI measurements: autonomic physiology and vigilance (as indexed with EEG) (Fig. 1). Our results confirm that both factors explain significant portions of fMRI variance, and demonstrate that the autonomic component of the fMRI signal increases as baseline vigilance decreases (Fig. 2). We find this relationship to be stronger during resting state than in a task with intermittent auditory stimuli, and to manifest most reliably in the default mode network, ventricles, and periventricular white matter. The spatiotemporal pattern of fMRI-autonomic correlations was largely consistent across baseline vigilance levels, albeit stronger and more widespread during low baseline vigilance, with early effects primarily localized in the grey matter and later effects in the ventricles and surrounding white matter (Fig. 3). More rapid EEG power changes (i.e., on the order of seconds) also exhibited stronger covariation with autonomic (Fig. 4) and fMRI signals (Fig. 5A–F) as baseline vigilance (i.e., minutes-long averages) decreased. Yet these effects could not fully explain (in a statistical sense) the changes in fMRI-autonomic covariance that we observed across more gradual shifts in baseline vigilance (Fig. 5G–I). In addition, we find that autonomic activity accounts for a large portion of vigilance-related fMRI functional connectivity (Fig. 6), which highlights the importance of modeling these time- and state-dependent sources of variance in neuroimaging analysis and interpretation (Chen et al., 2020;Gu et al., 2019).

### 4.1 Sites of vigilance-related covariance between fMRI and autonomic signals

Previous studies have reported spatial overlap between fMRI correlates of electrophysiological measures of vigilance and peripheral autonomic fluctuations (Gu et al., 2022;Özbay et al., 2019;Yuan et al., 2013). In addition, correlations between fMRI and EEG measures of vigilance appear weaker after removing autonomic fluctuations (Goodale et al., 2021;Yuan et al., 2013). These findings suggest that the effects of vigilance and autonomic activity on fMRI might covary. Our results extend these findings by relating baseline vigilance levels (indexed by EEG) to the proportion of fMRI variance explained by peripheral autonomic measures (Fig. 2). This relationship was widespread enough to manifest in the whole-brain average (i.e., global) fMRI signal (Fig. 2A), indicating that the autonomic contribution to the global signal changes with baseline vigilance. Yet it was also heterogenous, occurring most strongly in regions that have been associated with either vigilance (Goodale et al., 2021;X. Liu et al., 2018;Özbay et al., 2019;Soon et al., 2021;Yuan et al., 2013) or autonomic activity (Birn et al., 2006;Chang et al., 2009;Chen et al., 2020;Özbay et al., 2018,^2019^;Shmueli et al., 2007;Yuan et al., 2013): namely primary sensory (visual, motor, and auditory) cortices, brainstem, thalamus, precuneus, posterior cingulate cortex, orbitofrontal cortex, insula, amygdala, and hippocampus, along with the ventricles and periventricular white matter (Fig. 2C). Research on discrete vigilance conditions—such as eyes-closed versus eyes-open rest (Yuan et al., 2013) or high versus low task arousal (Roth et al., 2020)—has revealed greater autonomic effects in several of these areas during lower vigilance states, which our results replicate and extend to a broad continuum of windowed, baseline vigilance levels during both rest and task scans. In fact, our results remain largely consistent after excluding epochs likely to contain sleep (Supplementary Fig. S7), highlighting the continuous nature of fMRI-autonomic covariance across vigilance levels even when subjects were putatively awake.

While alert wakefulness and sleep may be readily distinguished from each other, measuring vigilance along a continuum is more complex (Merica & Fortune, 2004;Ogilvie, 2001). Though indirect, EEG is the most widely used and trusted method of detecting vigilance levels, usually involving a comparison of the power in middle (e.g., alpha) versus low (e.g., theta and/or delta) frequency bands (T. T. Liu & Falahpour, 2020;Merica & Fortune, 2004;Ogilvie, 2001). Behavioral measures, when available, often validate such EEG-based vigilance metrics by varying across changes in middle- and/or low-frequency electrophysiological power (Bodala et al., 2016;Cote et al., 2009;Ogilvie, 2001). Accordingly, our analysis involves indexing vigilance as the ratio of EEG power in the alpha versus theta bands (seeSection 2.4), and our task data indicate a strong relationship between this index and reaction times (Fig. 1B). To examine sensitivity to the specific choice of frequency bands, we also calculated a vigilance index that included delta-band power, and found that it yields highly similar results (Supplementary Fig. S5).

Since fMRI and autonomic signals have different temporal dynamics, determining the covariance between them requires accounting for this difference. In relating fMRI and autonomic measures at each baseline vigilance level, we used a basis set of response functions to model the relationship between autonomic and fMRI activity (seeSection 2.8). Although use of a basis set provides flexibility in the temporal mapping between signals, this procedure could also introduce a bias towards detecting fMRI-autonomic covariance that resembles the modeled functions. Yet a complementary approach based on cross-correlations—which evaluates relationships between signals at multiple lags without assuming a specific model—yielded results that were consistent with the basis function/regression approach. Specifically, cross-correlations revealed stronger fMRI-autonomic covariance during low baseline vigilance in the primary sensory cortices, brainstem, thalamus, precuneus, posterior cingulate cortex, ventricles, and periventricular white matter (Fig. 3).

Some of these structures, like the brainstem, are directly involved in the regulation of both autonomic activity and vigilance (Benarroch, 2017;Duyn et al., 2020;Iacovella & Hasson, 2011;Merica & Fortune, 2004;Silvani et al., 2015). For example, the brainstem’s preBötzinger complex controls breathing rhythms and behavioral arousal in mice, and projects to noradrenergic neurons in the locus coeruleus that convey its signals to the rest of the brain (Yackle et al., 2017). The parabrachial nuclear complex in the pons also controls breathing and vigilance, with projections to the basal forebrain, thalamus, hypothalamus, and amygdala (Shouman & Benarroch, 2021). Others, like the sensory cortices, may exhibit greater fMRI-autonomic covariance during lower vigilance via less direct mechanisms. In particular, neuronal excitability along sensory pathways decreases in drowsiness, resulting in fewer and smaller responses to the same stimuli in the sensory thalamic nuclei and primary sensory cortices (Coenen & Vendrik, 1972;Edeline et al., 2000;Livingstone & Hubel, 1981). Stronger fMRI-autonomic covariance in the primary sensory cortices during lower vigilance may therefore reflect down-regulated sensitivity to other events in these regions, which manifests as a greater share of fMRI variance attributable to autonomic fluctuations.

### 4.2 Temporal features of fMRI-autonomic covariance across EEG vigilance levels

While each autonomic measure had stronger and more spatially diffuse effects in fMRI data as baseline vigilance decreased, the spatiotemporal dynamics of fMRI-autonomic covariance were largely consistent across autonomic features (RV, HR, and PWA) and baseline vigilance levels (Fig. 3). The overall pattern of these dynamics exhibits earlier fMRI-autonomic covariance in the grey matter—especially the default mode and salience networks—and more delayed covariance in the ventricles and periventricular white matter, consistent with several previous reports (Birn et al., 2006;Chen et al., 2020;Gu et al., 2022;Özbay et al., 2018,^2019^;Shmueli et al., 2007;Yuan et al., 2013).

These fMRI-autonomic correlations may arise from systemic and/or neuronal vascular effects, as many of the strongest relationships between fMRI and autonomic signals occur in areas with high vascular density (Bernier et al., 2018). Additionally, the temporal dynamics of fMRI-autonomic correlations bear some resemblance to local differences in blood transit times (as measured with intravenous, paramagnetic contrast agents), which tend to be 4 to 8 s slower in areas of higher venous density than in those of higher arterial density (Tong et al., 2017). Vascular reactivity may also be as much as 4 s slower in posterior, subcortical, and superior midline areas (Chang et al., 2008), including those with some of the strongest and most delayed covariations between fMRI and autonomic signals. Time-delayed effects, such as the negative correlations between RV or HR activity and widespread fMRI signals 8 to 12 s later, could also reflect vascular mechanisms, as changes in the vascular concentration of carbon dioxide are closely associated with respiratory and cardiac fluctuations and with fMRI effects around these latencies (Chang & Glover, 2009;Nakada et al., 2001;Thomason et al., 2005). Yet whether these effects arise from systemic physiological and/or neurogenic processes, like autonomic regulation or changes in neuronal excitability, remains unclear. Understanding what drives changes in fMRI-autonomic covariance across vigilance levels will therefore require closer inspection and comparison of these potential mechanisms.

Here, we defined baseline vigilance levels by averaging EEG spectral features over minutes-long windows. Yet since electrophysiological power also fluctuates within these long windows (seeFig. 1A)—especially during states of lower vigilance (Hertig-Godeschalk et al., 2020;X. Liu et al., 2018;Soon et al., 2021)—we investigated whether more rapidly varying (“fast”) EEG features might also covary with autonomic and/or fMRI signals. When sampled on the level of seconds, the ratio of EEG power in the alpha versus theta bands exhibited moderate correlations with autonomic signals that strengthened as baseline vigilance (i.e., the average alpha/theta ratio across minutes) decreased (Fig. 4). This finding is consistent with previous reports of stronger correlations between alpha power and RVT during eyes-closed versus eyes-open rest (Yuan et al., 2013) and of large correlations between low-frequency electrophysiological power (i.e., 0.5–2 Hz) and various autonomic measures during light sleep (Özbay et al., 2019;Picchioni et al., 2022), microsleeps (Soon et al., 2021), and low-vigilance states (X. Liu et al., 2018). These correlations suggest that the fMRI-autonomic covariance effects discussed above may coincide with or even arise from the same processes that drive covariance between fMRI and relatively fast EEG signals. Accordingly, we observed stronger relationships between fMRI and seconds-level EEG signals as baseline vigilance waned. Yet autonomic variance could not account for these fMRI-fast EEG effects, nor could the variance of seconds-level EEG signals account for the changes in fMRI-autonomic covariance across baseline vigilance levels (Fig. 5). Fast electrophysiological activity indicative of neuronal population firing, and autonomic fluctuations indicative of neuronal and/or vascular phenomena, while sharing some proportion of variance with one another, may also have unique influences on fMRI signals as baseline vigilance levels shift.

However, EEG signals acquired during fMRI scanning tend to have lower signal-to-noise ratios than physiological recordings, due to artifacts of the MRI environment that may not be fully removed during preprocessing. In addition, the basis sets used here to model the effects of fast EEG and autonomic signals in fMRI data resulted in nine EEG regressors and 13 autonomic ones, which could have biased the amount of variance explained by each measure even though we adjusted the coefficients of determination (R^2^s) accordingly for the comparison of variance explained by each. Evaluating covariance with fMRI signals also depends largely on the transfer function used to model the relationship between each measure and brain hemodynamics. Here, we used two basis sets for the fast EEG-fMRI convolution: one derived from the canonical hemodynamic response function and another based on the cross-correlation between fast EEG and fMRI signals as well as previous research (de Munck et al., 2007). For autonomic-fMRI convolutions, we used basis sets derived from the canonical hemodynamic response function (for PWA) and previously defined respiratory and cardiac response functions (Birn et al., 2008;Chang et al., 2009;Chen et al., 2020). While each of these approaches is grounded in prior research, it is possible that different, for example, more complex models may better represent the covariance between these measures and fMRI signals (cf.Mann-Krzisnik & Mitsis, 2022). Accordingly, determining how EEG power and autonomic fluctuations influence each other and fMRI signals over timescales of seconds requires further research.

While many of our analyses employ sliding windows of 126 s each to stage baseline vigilance levels, the brain regions that we find to exhibit more fMRI-autonomic covariance with gradual decreases in baseline vigilance closely resemble those implicated in briefer (i.e., <4 s) vigilance fluctuations (Goodale et al., 2021;Özbay et al., 2019;Raut et al., 2021;Soon et al., 2021;Yuan et al., 2013), suggesting that the same mechanisms may be involved in vigilance-related changes across different timescales. Consistent with this interpretation, our fMRI-autonomic covariance analyses also arrived at these regions with two very different window lengths of 126 and 241.5 s each (seeFig. 2andSupplementary Fig. S4, and tests of more window lengths inSupplementary Fig. S3B). The similarities between vigilance effects on the orders of seconds and minutes also raise the possibility that a few short events, like microsleeps (Hertig-Godeschalk et al., 2020;Soon et al., 2021) or transient arousal shifts (Gu et al., 2019;X. Liu et al., 2018), could drive much of the variance in relatively long windows. Microsleeps are also found to be longer and more common as one’s overall vigilance wanes (Hertig-Godeschalk et al., 2020;Soon et al., 2021). The generally continuous relationship that we observe between minutes-long EEG vigilance levels and fMRI-autonomic covariance could thus arise from gradually more microsleeping across the spectrum of high-to-low baseline vigilance, along with accompanying fMRI and autonomic changes (Hertig-Godeschalk et al., 2020;Soon et al., 2021). Further research may identify instances of sleep events and sleep stages to investigate their specific contribution to the present results. More work is also needed to elucidate whether and how vigilance-related effects in fMRI signals differ across timescales.

### 4.3 Vigilance effects on functional connectivity

Functional connectivity (FC) analysis is a prevalent technique for understanding brain organization across individuals (J. Zhang et al., 2021) with close links to both vigilance (Wang et al., 2016) and autonomic measures (Chen et al., 2020;Xifra-Porxas et al., 2021). To explore how changes in fMRI-autonomic covariance across vigilance levels might affect the results of this widely used approach, we assessed the FC between canonical brain networks across baseline vigilance levels with and without autonomic variance partialled out. Before removing autonomic fluctuations, we found widespread vigilance-associated FC effects in resting state, with fewer effects during the vigilance-probing task (especially when we included stimulus regressors in this analysis) (Fig. 6A–C). Previous studies have described a negative relationship between vigilance and the FC of the default mode with the dorsal and ventral attention networks, the subcortex with the ventral attention/salience network, and the control with the dorsal and ventral attention networks (Chang et al., 2013;Larson-Prior et al., 2011;Sämann et al., 2011;Wang et al., 2016). Our findings corroborate these results and extend them to include evidence of default mode-somatomotor network FC decreasing with baseline vigilance, and subcortical-ventral attention/salience network FC decreasing with baseline vigilance during rest but increasing with vigilance during a psychomotor task (when not including stimulus regressors in the analysis). We also found vigilance-associated effects of limbic FC, but these merit extra caution given the percentage of limbic voxels (5% from resting-state and 23% from task) that we excluded from our analyses due to missing data (seeSection 2.2;Supplementary Table S1;Supplementary Fig. S8).

Removing the variance associated with autonomic fluctuations had little effect on the relationship between baseline vigilance and FC in the auditory task examined here, especially when we had already removed the variance associated with the stimuli (Fig. 6D–E). Yet accounting for autonomic signals significantly altered resting-state results (Fig. 6F). For example, increased baseline vigilance was associated with increased FC of the dorsal attention network with the ventral attention/salience and somatomotor networks, implying greater cohesion between them commensurate with their roles in monitoring and responding to one’s environment (Corbetta & Shulman, 2002). These correlations are consistent with those reported byWang et al. (2016)after regressing out global, white matter, and cerebrospinal fluid fMRI signals—which we (Fig. 3) and others (e.g.,Xifra-Porxas et al., 2021) have shown to be closely related to autonomic fluctuations. More broadly, the extent of the changes in vigilance-associated effects after removing autonomic fluctuations underscores the close tie between these phenomena, particularly during rest.

Yet, though effects in resting-state scans tended to be larger, vigilance-related changes in network-to-network FC tended to follow a similar pattern during task and rest scans (Fig. 6G, I). Regressing out autonomic fluctuations considerably strengthened this task-rest correspondence when stimulus effects were not partialled out of the task data (Fig. 6J), but had a negligible impact when controlling for stimulus effects (Fig. 6D). This difference is likely due to the close correlation between the stimulus regressors and autonomic signals (seeSection 3.2), such that removing the variance associated with task stimuli probably also removed much of the autonomic variance from the task scans. Similarly, the correlations between baseline vigilance and fMRI-autonomic covariance might be weaker when including stimulus regressors (Fig. 2;Table 1) because removing autonomic variance also reduces fMRI-autonomic covariance.

### 4.4 Differences between rest and task conditions

While including stimulus regressors might have removed a significant portion of autonomic and fMRI variance, vigilance-related effects during the task condition were consistently weaker even without these regressors. In the case of the limbic network, this could be due to variance in participants’ positions relative to the fMRI acquisition fields of view, which resulted in more voxels missing data during the task condition (23.08%) than during resting-state scans (4.88%) (Supplementary Table S1;Supplementary Fig. S8). In other regions, such as those involved in auditory and motor processing, the neural activity associated with listening and responding to occasional tones likely reduced the proportion of fMRI variance shared with autonomic activity. One potential explanation is that the psychomotor vigilance task requires more alertness than rest, reducing the variability of vigilance and/or autonomic activity.Wang et al. (2016)found greater correspondence between rest and task FC during high vigilance, and the EEG vigilance levels that we measured were generally less variable during the task (mean ± S.D. alpha/theta ratio = 1.08 ± 0.42) than rest (1.05 ± 0.51), though they spanned a wider range of values (task range = 0.44–3.00, rest range = 0.50–2.14). Yet even when we restricted task data to include only sliding windows that had baseline vigilance levels within the range of those from the rest condition, the vigilance-related effects of fMRI-autonomic covariance were virtually unchanged (Supplementary Fig. S9).

The task stimuli also likely contributed to the differences between vigilance effects in the two conditions. The sparse and randomly spaced auditory tones may have elicited rapid autonomic activity like orienting responses (seeDampney, 2015;Hilton, 1982) that effectively decoupled autonomic signals from gradual changes in EEG vigilance. Moreover, if some stimuli were more alerting than others (e.g., those presented during lower vigilance), they might have evoked different autonomic (McDonald et al., 1964) and fMRI responses (Roth et al., 2020) which our binary “on” or “off” stimulus covariates failed to capture (seeSection 2.8). Stimuli being more alerting during lower baseline vigilance levels might also explain why HR variance increased as baseline vigilance waned during the task. These possibilities underscore the importance of preprocessing and statistical modeling in the analysis and interpretation of the complex and interrelated phenomena that underlie fMRI measurements (T. T. Liu, 2016;Xifra-Porxas et al., 2021). Yet distinguishing the precise effects of stimuli—especially from those of vigilance changes—could prove difficult given the propensity for stimuli to influence alertness and evoke the neural and autonomic responses that accompany it (Dampney, 2015;Hilton, 1982;McDonald et al., 1964;Roth et al., 2020). Accordingly, a systematic investigation of stimulus-evoked vigilance and autonomic responses may be able to shed more light on how to best model these effects in fMRI data. Assessing larger samples of participants might also help to understand how the relationships between vigilance, fMRI variance, autonomic signals, and modeling differ between task and rest conditions. Though many of the effects that we observed are strong and robust to permutation tests and multiple comparisons correction, larger samples may inform the generalizability of our results.

### 4.5 Implications for fMRI data analysis and interpretation

Here, a systematic analysis across EEG vigilance levels reveals that fMRI signals exhibit an increasing proportion of autonomic variance as baseline vigilance wanes. This change in fMRI-autonomic covariance, including its spatiotemporal dynamics and its influence on measures of functional connectivity, has several implications on the analysis and interpretation of fMRI data.

On the one hand, it is common to “denoise” fMRI data of autonomic variance and its correlates (e.g., the global fMRI signal, or signals derived from the white matter and ventricles) (Behzadi et al., 2007;Salimi-Khorshidi et al., 2014) to improve the quantification of BOLD responses arising from local neural activity (reviewed inDuyn et al., 2020;T. T. Liu, 2016). Our results imply that doing so would remove more variance within periods of lower vigilance—especially in the default mode network, ventricles, and periventricular white matter—which may or may not be the intended effect. Some analyses could thus benefit by accounting for dynamic vigilance levels and their effects on fMRI-autonomic covariance. Even when direct vigilance measures are unavailable, data-driven methods have shown great promise in decoding vigilance (Falahpour et al., 2018;Goodale et al., 2021;Wang et al., 2016) and autonomic (Aslan et al., 2019;Bayrak et al., 2021;Salas et al., 2021) information from fMRI measures alone.

Yet on the other hand, our findings suggest that removing autonomic signals from fMRI data could also remove electrophysiological-, hemodynamic-, and/or vigilance-related signals. Some cognitive, emotional, or other processes might consequently be more detectable when autonomic variance is retained—especially in data from tasks that elicit correlated autonomic and neural activity. Autonomic measures can also offer valuable insights into brain organization and function themselves (see, e.g.,Bright et al., 2020;Mather & Thayer, 2018;Shokri-Kojori et al., 2018). Accordingly, some evidence suggests that vascular regulation operates in concert with and in support of neuronal function (Bright et al., 2020;J. H. Zhang et al., 2012). Consistent with this hypothesis, cerebrovascular reactivity has been linked to cognitive function, for example, in Alzheimer’s disease (Cantin et al., 2011;Kong et al., 2020), and cardiac vagal control has been implicated in depression (Stapelberg et al., 2012). Changes in autonomic and fMRI variance, such as those that we observe across baseline vigilance levels, might therefore reflect cerebrovascular phenomena that underlie brain function, rather than “noise” to be discarded. Indeed, removing autonomic variance from the fMRI signals of Alzheimer’s patients makes them harder to distinguish from healthy controls (Li et al., 2021), and may likewise conceal a wide range of functional information—like vigilance-associated operations—in these and other data.

Ultimately, the best way to handle autonomic and fMRI signals depends on the research question at hand. Our findings shed new light on the temporal variability of these data, and indicate that vigilance is a significant factor to consider when analyzing and interpreting them. Accordingly, the mechanisms through which vigilance, autonomic fluctuations, and neural activity interact merit further research as we advance our understanding of brain function and dysfunction.

## Data and Code Availability

Code is publicly available athttps://osf.io/3a2ut/. Data are available upon request without restriction, and will be posted to a database (with a link provided from the aforementioned OSF site) pending approval from the local ethics committee.

## Author Contributions

Benjamin P. Gold: Data curation, Formal analysis, Investigation, Methodology, Project administration, Visualization, Writing—original draft, and Writing—review & editing. Sarah E. Goodale: Data Curation, Software, and Writing—review & editing. Chong Zhao: Investigation, Writing—review & editing. Haatef Pourmotabbed: Investigation, Writing—review & editing. Jacco A. de Zwart: Investigation, Writing—review & editing. Pinar S. Özbay: Investigation, Writing—review & editing. Taylor S. Bolt: Investigation, Writing—review & editing. Jeff H. Duyn: Resources, Investigation, and Writing—review & editing. Jingyuan E. Chen: Investigation, Writing—review & editing. Catie Chang: Conceptualization, Formal analysis, Methodology, Writing—original draft, Writing—review & editing, Project administration, and Funding acquisition.

## Funding

This work was supported by National Institutes of Health grants T32 EB001628 (Benjamin P. Gold), K22 ES028048, RF1 MH125931, and P50 MH109429 (Catie Chang), and F99 AG079810 and a National Science Foundation GRFP (Sarah E. Goodale). This work was also supported in part by the Intramural Research Program of the National Institute of Neurological Disorders and Stroke.

## Ethics Statement

All participants gave written informed consent, and ethical approval was granted by the Institutional Review Boards of the National Institutes of Health (Protocol 00-N-0082) and Vanderbilt University (IRB #181540).

## Declaration of Competing Interest

The authors declare no conflict of interest.

## Supplementary Material

Supplementary Material
