# Supplementary material for: Functional MRI signals exhibit stronger covariation with peripheral autonomic measures as vigilance decreases
Source: Imaging Neurosci (Camb). 2024 Sep 13;2:imag-2-00287. doi: 10.1162/imag_a_00287 (PMC12290586; doi:10.1162/imag_a_00287)
Supplement: Supplementary Material [file imag_a_00287-supp.pdf]

SUPPLEMENTARY MATERIAL

Table S1. Percentage of voxels with missing data in each canonical network

Network	Resting-state participants	Task participants
Cont	0.09	0.03
Default	0.06	0.53
Dors Att	0	8.45
Limb	4.88	23.08
Sal/Vent Att	0	0
Somatomotor	0	3.88
Visual	0	0.17
Subcortex	0	0

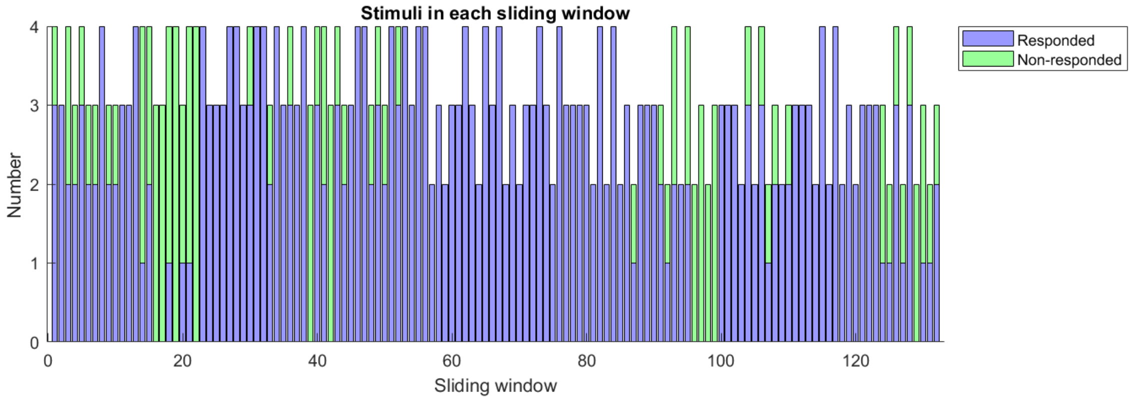

Figure S1: Stimuli and responses in each 126-s sliding window. Dividing the psychomotor vigilance task into sliding windows of 60 TRs (126 s) each resulted in windows with 2 - 4 stimuli (mean ± S.D. = 3.15 ± 0.73) and 0 - 4 responses (mean ± S.D. = 2.48 ± 1.14).

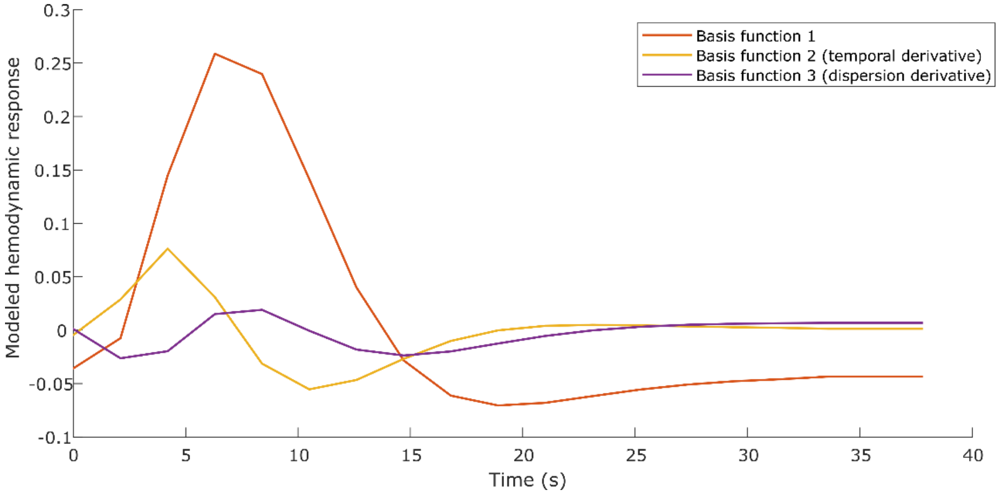

Figure S2: Basis set of hemodynamic responses to fast EEG activity derived from fMRI-EEG cross-correlations. We modeled the transfer between fast, seconds-level EEG signals and fMRI responses with a basis set focused on a similar lag and dispersion to the cross-correlations that we observed between these data (see Figure S6). We then convolved the seconds-level EEG alpha, theta, and delta power (sampled every 2.1 s) with this basis set to evaluate the covariance shared between the EEG and fMRI signals in different tissue types (see Figure 5D - F).

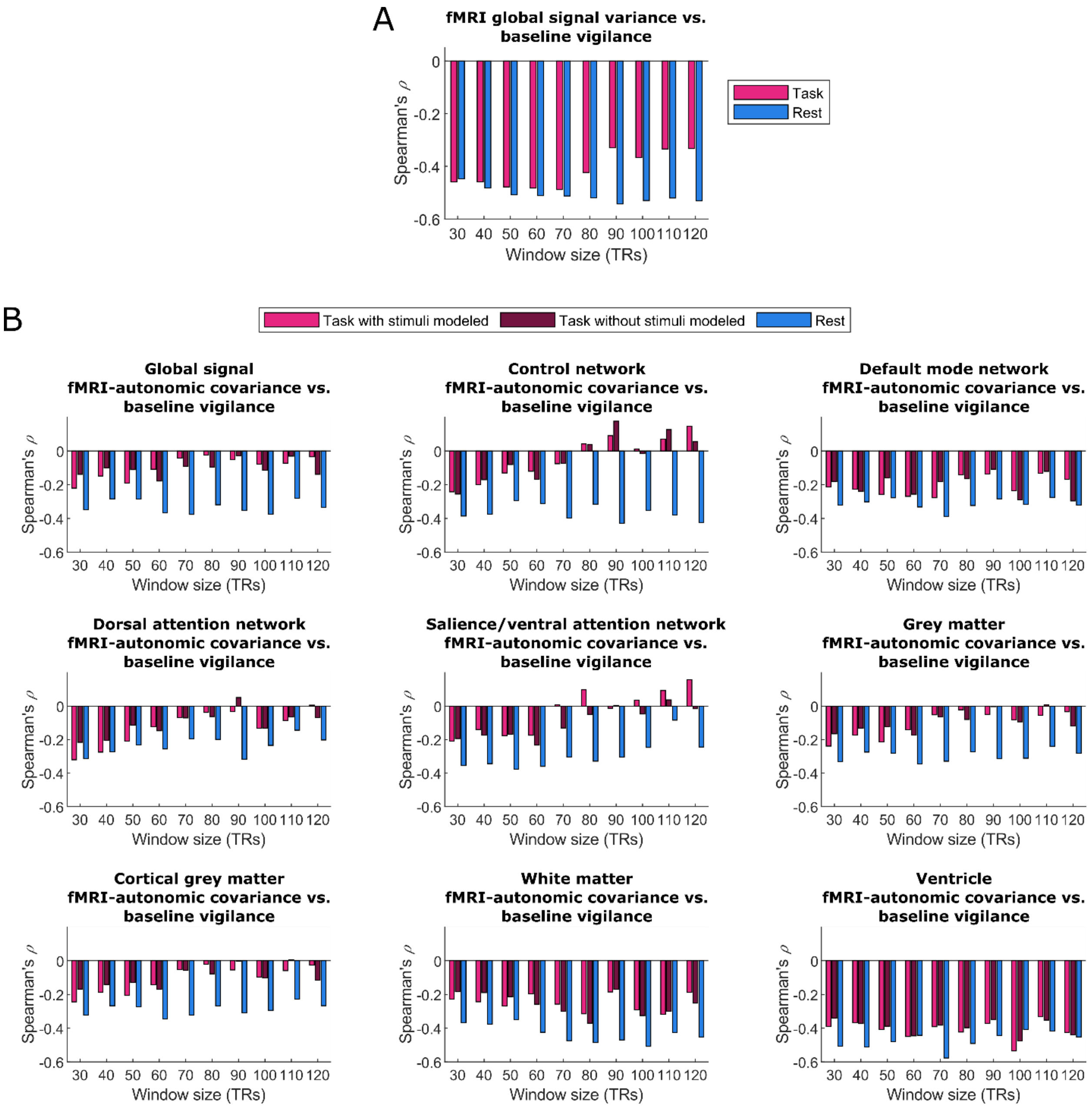
A
fMRI global signal variance vs. baseline vigilance
Spearman's ρ
Window size (TRs)
Task
Rest
B
Task with stimuli modeled
Task without stimuli modeled
Rest
Global signal fMRI-autonomic covariance vs. baseline vigilance
Control network fMRI-autonomic covariance vs. baseline vigilance
Default mode network fMRI-autonomic covariance vs. baseline vigilance
Dorsal attention network fMRI-autonomic covariance vs. baseline vigilance
Salience/ventral attention network fMRI-autonomic covariance vs. baseline vigilance
Grey matter fMRI-autonomic covariance vs. baseline vigilance
Cortical grey matter fMRI-autonomic covariance vs. baseline vigilance
White matter fMRI-autonomic covariance vs. baseline vigilance
Ventricle fMRI-autonomic covariance vs. baseline vigilance

Figure S3: Vigilance-related changes in fMRI signal variance and fMRI-autonomic covariance across different window sizes. Most of our analyses used windows of 126 s (60 TRs) each to limit the amount of vigilance states within each window while still capturing sufficient data for each one. Yet this selection could influence our results, and so we evaluated vigilance-related changes in **(A)** the variance of the fMRI global signal and **(B)** in fMRI-autonomic covariance in the global signal and certain key networks across windows from 63 s (30 TRs) to 252 s (120 TRs) to assess the robustness of our main findings. This analysis revealed relatively stable results for resting-state data, and for task data for windows of about 63 to 147 s each. In longer windows of the task data, fMRI global signal variance and fMRI-autonomic covariance – especially in the control, salience/ventral attention, and dorsal attention networks – exhibited less negative/more positive associations with baseline vigilance. Since the task stimuli occurred at up to 88.76 s apart, and since these brain networks are involved in monitoring for and planning responses to stimuli, we speculate that the demands and stimuli of the psychomotor vigilance task may have increased the number of vigilance states sampled in these long task windows, thereby disrupting the relationship observed with shorter windows.

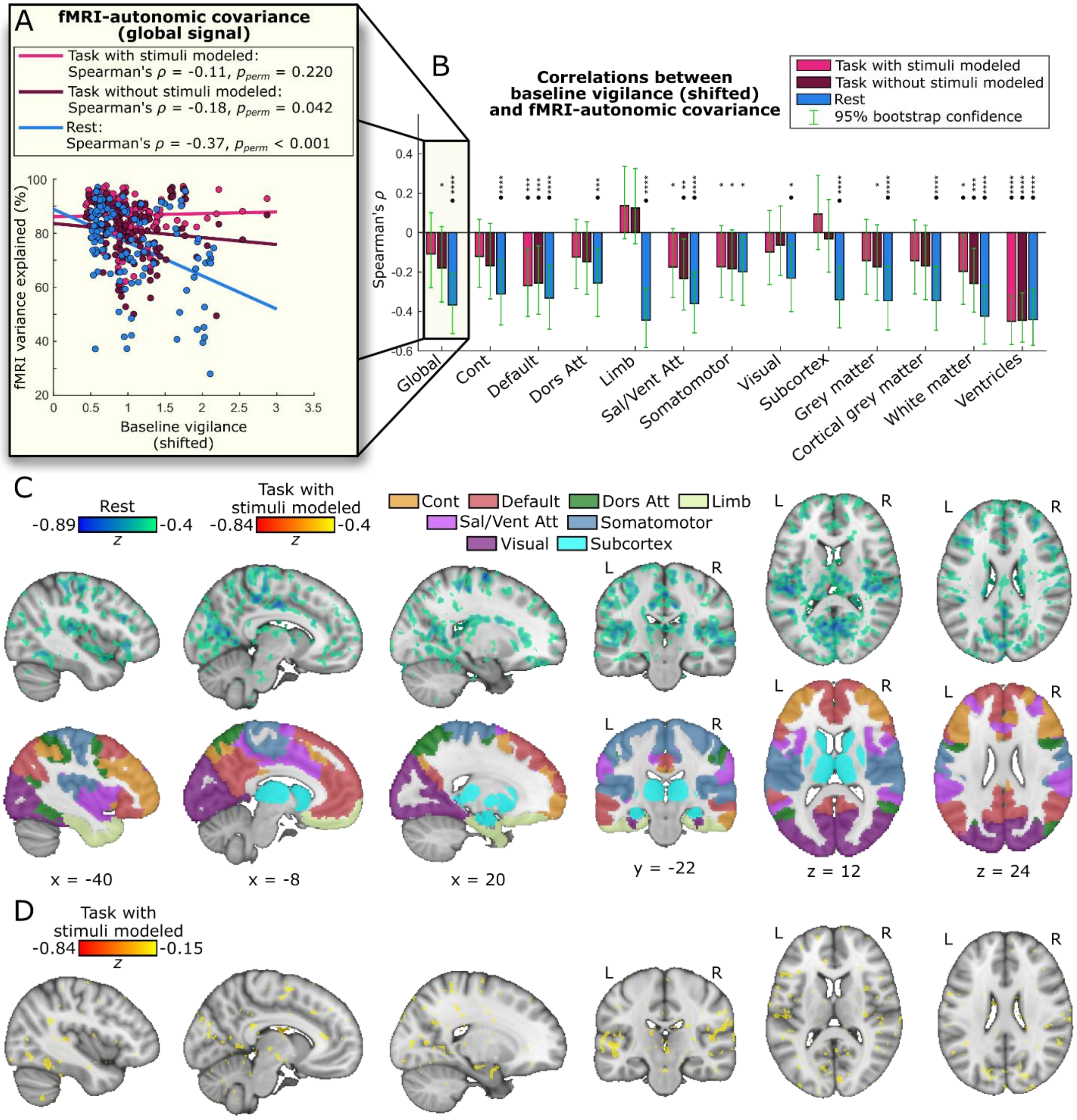
A
fMRI-autonomic covariance (global signal)
Task with stimuli modeled: Spearman's ρ = -0.11, p_perm = 0.220
Task without stimuli modeled: Spearman's ρ = -0.18, p_perm = 0.042
Rest: Spearman's ρ = -0.37, p_perm < 0.001
fMRI variance explained (%)
Baseline vigilance (shifted)
B
Correlations between baseline vigilance (shifted) and fMRI-autonomic covariance
Task with stimuli modeled
Task without stimuli modeled
Rest
95% bootstrap confidence
Spearman's ρ
Global
Cont
Default
Dors Att
Limb
Sal/Vent Att
Somatomotor
Visual
Subcortex
Grey matter
Cortical grey matter
White matter
Ventricles
C
Rest
-0.89
-0.4
z
Task with stimuli modeled
-0.84
-0.4
z
Cont
Default
Dors Att
Limb
Sal/Vent Att
Somatomotor
Visual
Subcortex
L
R
x = -40
x = -8
x = 20
y = -22
z = 12
z = 24
D
Task with stimuli modeled
-0.84
-0.15
z

Figure S4: Vigilance-related changes in voxelwise fMRI-autonomic covariance with windows of 60 TRs (126 s) each. We assessed fMRI-autonomic covariance across baseline vigilance levels in each voxel. While we originally used windows of 115 TRs (241.5 s) to accommodate the noisiness of fMRI signals (see Figure 2), we tested the robustness of these effects by reevaluating them in windows of 60 TRs (126 s), to match those for the global- and network-based analyses. The resulting effects were largely similar to those observed with 115-TR windows for **(A)** the global signal (visualized here with least-squares trend lines although the correlations are based on non-parametric statistics) and **(B)** the networks of interest. **(C)** Comparing the voxelwise results to our original results indicated slightly more widespread effects during resting state and sparser during the psychomotor vigilance task. However, lowering the visualization threshold from -0.4 to -0.15 revealed a similar pattern of correlations in the task condition to those observed with 241.5-s windows. Global = global signal, Cont = control network, Default = default mode network, Dors Att = dorsal attention network, Limb = limbic network, Sal/Vent Att = salience/ventral attention network, Somatomotor = somatomotor network, Visual = visual network, * = $p_{perm} \leq 0.05$, ** = $p_{perm} \leq 0.01$, *** = $p_{perm} \leq 0.005$, **** = $p_{perm} \leq 0.001$, • = survives multiple-comparisons correction with a false discovery rate of 5%.

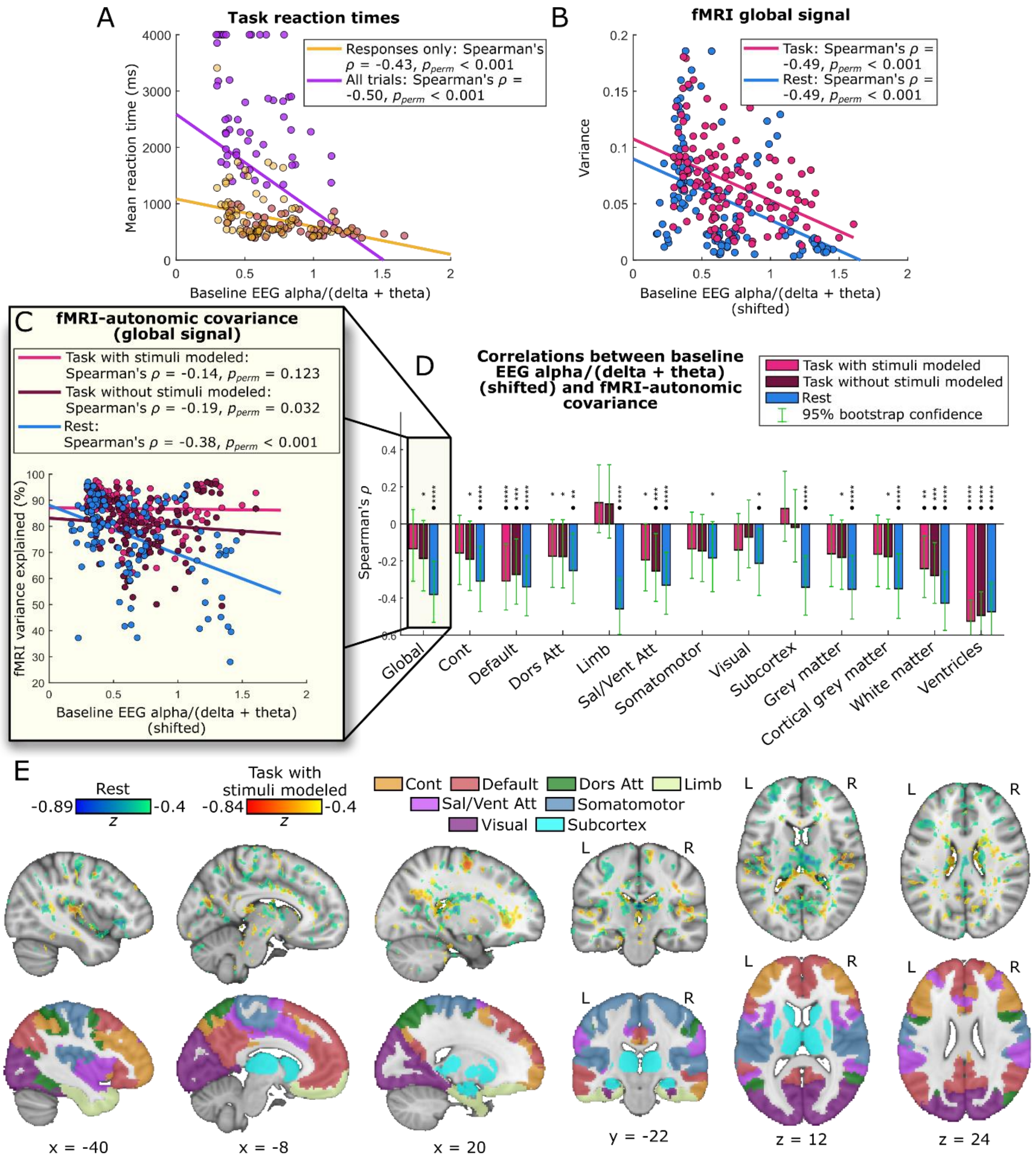
A
Task reaction times
Responses only: Spearman's ρ = -0.43, pperm < 0.001
All trials: Spearman's ρ = -0.50, pperm < 0.001
Mean reaction time (ms)
Baseline EEG alpha/(delta + theta)
B
fMRI global signal
Task: Spearman's ρ = -0.49, pperm < 0.001
Rest: Spearman's ρ = -0.49, pperm < 0.001
Variance
Baseline EEG alpha/(delta + theta) (shifted)
C
fMRI-autonomic covariance (global signal)
Task with stimuli modeled: Spearman's ρ = -0.14, pperm = 0.123
Task without stimuli modeled: Spearman's ρ = -0.19, pperm = 0.032
Rest: Spearman's ρ = -0.38, pperm < 0.001
fMRI variance explained (%)
Baseline EEG alpha/(delta + theta) (shifted)
D
Correlations between baseline EEG alpha/(delta + theta) (shifted) and fMRI-autonomic covariance
Task with stimuli modeled
Task without stimuli modeled
Rest
95% bootstrap confidence
Spearman's ρ
Global
Cont
Default
Dors Att
Limb
Sal/Vent Att
Somatomotor
Visual
Subcortex
Grey matter
Cortical grey matter
White matter
Ventricles
E
Rest
-0.89
-0.4
z
Task with stimuli modeled
-0.84
-0.4
z
Cont
Default
Dors Att
Limb
Sal/Vent Att
Somatomotor
Visual
Subcortex
L
R
x = -40
x = -8
x = 20
y = -22
z = 12
z = 24

Figure S5: Vigilance-associated effects when using a different baseline vigilance index. Since different studies have used different frequency bands to measure EEG vigilance, we recalculated the relationships between baseline vigilance levels and **(A)** task reaction times, **(B)** variance in the fMRI global signal, and **(C - E)** fMRI-covariance using another common metric: the ratio of power in the alpha band (8 - 12 Hz) vs. that in the delta and theta bands (0.5 - 7 Hz). Using this ratio yielded highly similar results to those obtained by using the alpha/theta ratio (see Figure 1B - C and Figure 2), with only minor changes to the coefficients and p_{perm} values of these effects that had no bearing on statistical significance. Although these correlations are based on non-parametric statistics, we include least-squares trend lines for visualization. Global = global signal, Cont = control network, Default = default mode network, Dors Att = dorsal attention network, Limb = limbic network, Sal/Vent Att = salience/ventral attention network, Somatomotor = somatomotor network, Visual = visual network, * = $p_{perm} \leq 0.05$, ** = $p_{perm} \leq 0.01$, *** = $p_{perm} \leq 0.005$, **** = $p_{perm} \leq 0.001$, • = survives multiple-comparisons correction with a false discovery rate of 5%.

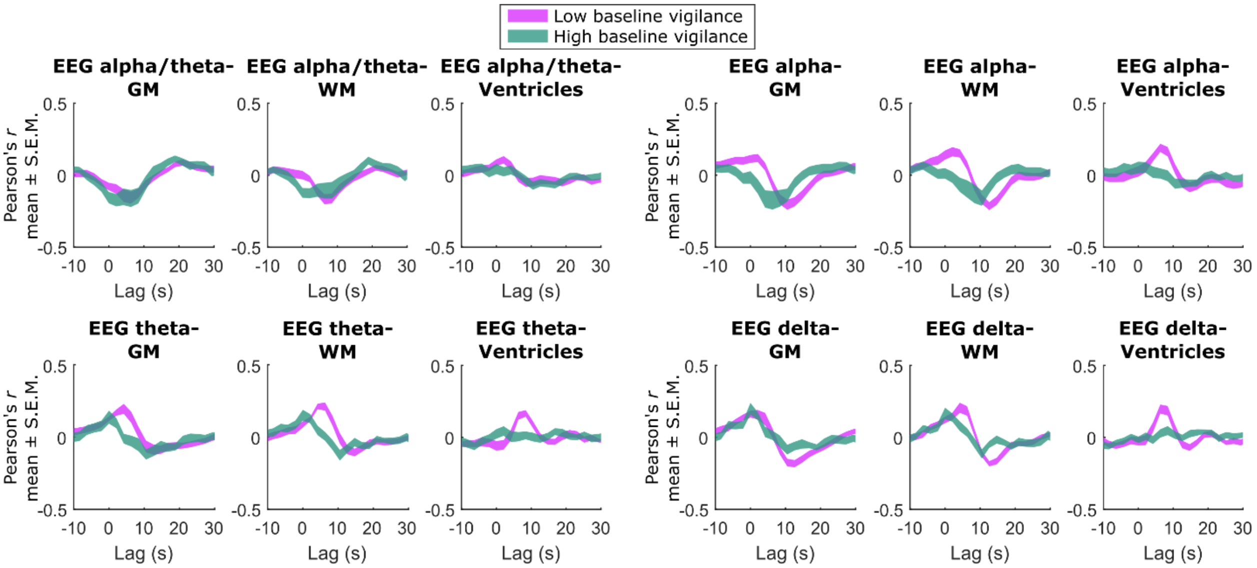

Figure S6: Spatiotemporal dynamics of fMRI-fast EEG correlations in low and high baseline vigilance across voxels and tissue types. To identify where and when fMRI signals correlated with fast, seconds-level EEG signals during periods of low vs. high baseline (i.e., minutes-level) EEG vigilance, we calculated the mean Pearson correlation between the fMRI signal in each voxel (sampled at the TR of 2.1 s) and each EEG signal (also sampled every 2.1 s) during the 2.1-min sliding windows with the lowest and highest EEG vigilance levels, at lags from -10.5 to 31.5 seconds. We then averaged the voxelwise values separately for grey matter (GM), white matter (WM), and ventricles to compare the effects in low vs. high baseline vigilance. Curves illustrating the range of mean ± the standard error of the mean (S.E.M.) for each cross-correlations show that fMRI-fast EEG correlations were generally larger and more delayed during low vs. high baseline vigilance, especially in the ventricles and in the alpha and delta frequency bands.

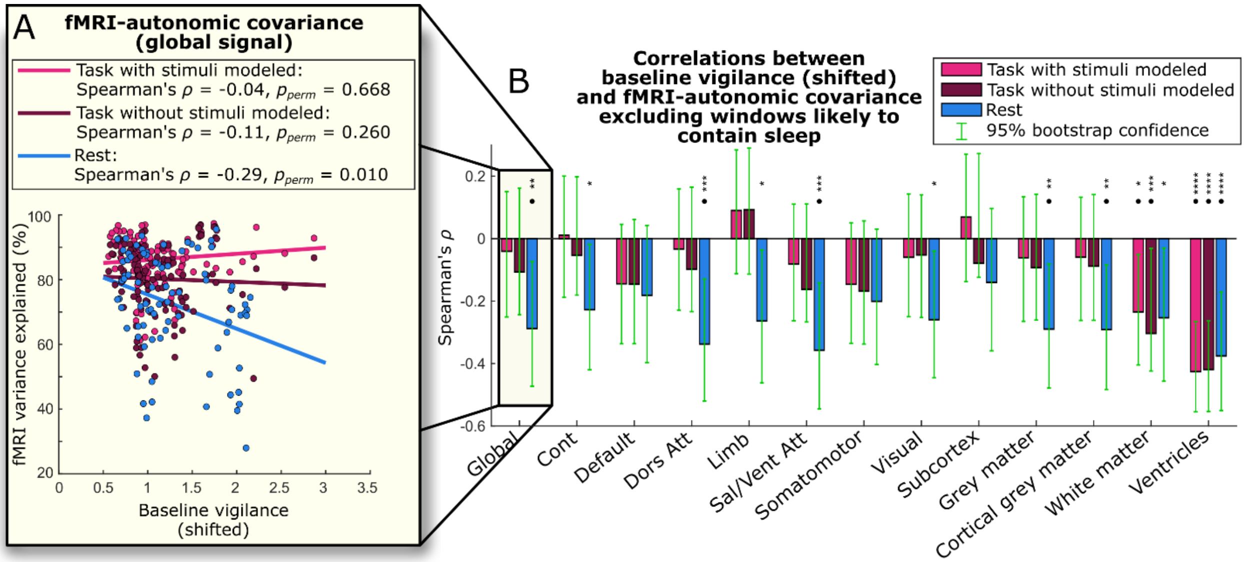

Figure S7: Relationships between baseline vigilance and fMRI-autonomic covariance when excluding task windows likely to contain sleep. The correlation between baseline vigilance levels and fMRI-autonomic covariance may be a continuous effect, such that autonomic signals explain progressively more variance in fMRI data as vigilance wanes, or may reflect a strong influence of sleep and its associated physiological changes. We explored this latter possibility by conducting the same linear regressions between window-averaged, baseline vigilance levels and global/network fMRI-autonomic covariance as in Figure 2A - B, but this time excluding the 11/132 windows from the psychomotor vigilance task during which participants failed to respond to the stimuli and the 40/121 windows with the lowest baseline vigilance levels during resting state. **(A)** Whole-brain fMRI-autonomic covariance exhibits weak, non-significant negative correlations with baseline vigilance during the task, and significant negative correlations during resting state, similar to the effects we observed across all baseline vigilance levels in Figure 2A. Although these correlations are based on non-parametric statistics, we include least-squares trend lines for visualization. For the task data with stimulus covariates, this gives a positive slope on the best-fitting line despite a negative Spearman correlation. **(B)** Conducting this analysis in predefined brain networks indicates that excluding sleep epochs generally weakens the effects, especially in grey matter, although all results trend in the same direction as in Figure 2B. These results could reflect a driving role of sleep in the relationship between vigilance and grey-matter fMRI-autonomic covariance, or a drop in statistical power due to the lower number of windows in these analyses. Global = global signal, Cont = control network, Default = default mode network, Dors Att = dorsal attention network, Limb = limbic network, Sal/Vent Att = salience/ventral attention network, Somatomotor = somatomotor network, Visual = visual network, * = $p_{perm} \leq 0.05$, ** = $p_{perm} \leq 0.01$, *** = $p_{perm} \leq 0.005$, **** = $p_{perm} \leq 0.001$, • = survives multiple-comparisons correction with a false discovery rate of 5%.

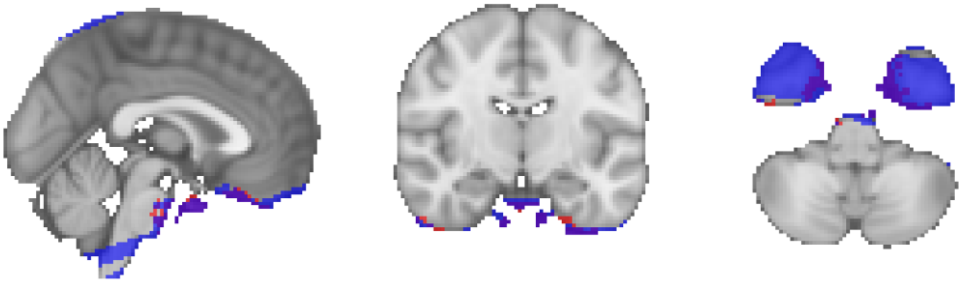

Figure S8: Voxels with missing data during the task and resting-state scans. Limited fields of view occasionally resulted in voxels missing data from one or more participant; we therefore excluded these voxels from all analyses. Here, we show the voxels that had missing data during the task scans (in blue) and resting-state scans (in red) in radiological convention at MNI coordinates x = 0, y = -12, z = -44.

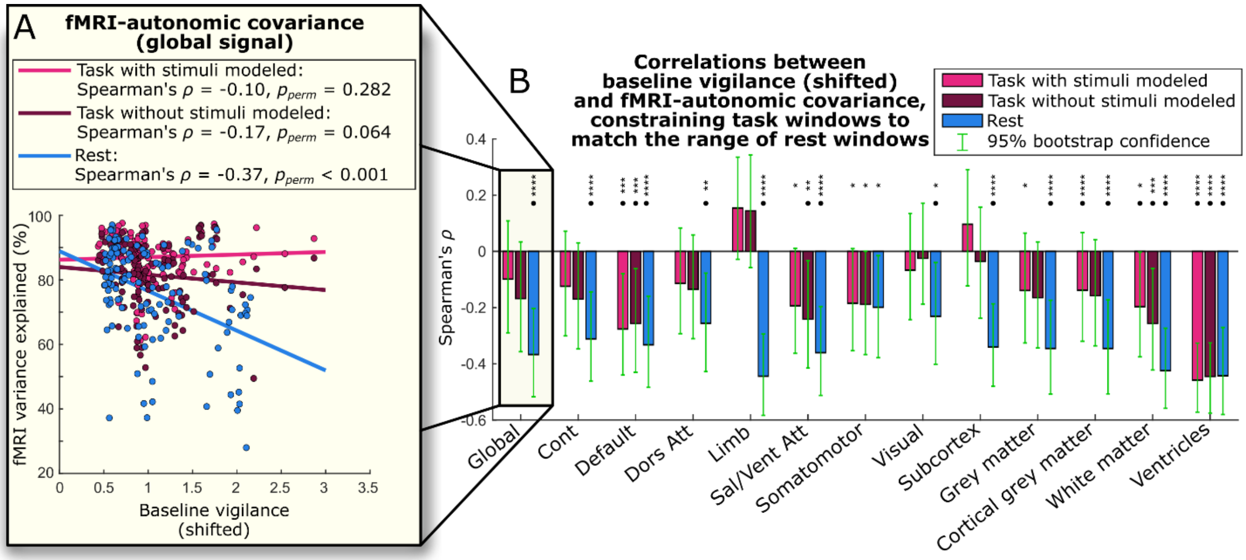

Figure S9: Relationships between baseline vigilance and fMRI-autonomic covariance when task and rest data share similar baseline vigilance ranges. Across the sliding windows of 126 s each, task baseline vigilance levels ranged from alpha/theta ratios of 0.44 to 3.00, whereas rest levels ranged from 0.53 to 2.01. Differences between task and resting-state results may therefore arise from different baseline vigilance ranges. We examined this possibility by conducting the same linear regressions between baseline vigilance levels and global/network fMRI-autonomic covariance as in Figure 2A - B, but this time excluding the task windows with greater or lesser baseline vigilance levels than those of the resting-state windows. **(A)** Whole-brain fMRI-autonomic covariance during the psychomotor vigilance task exhibits weak, non-significant negative correlations with baseline vigilance, similar to the effects we observed across all task baseline vigilance levels in Figure 2A. Although these correlations are based on non-parametric statistics, we include least-squares trend lines for visualization. **(B)** Conducting this analysis in predefined brain networks reveals a highly similar pattern of results as in Figure 2B, indicating that the relationship between baseline vigilance and fMRI-autonomic covariance during the task is not due to the wider range of baseline vigilance levels in this condition. Global = global signal, Cont = control network, Default = default mode network, Dors Att = dorsal attention network, Limb = limbic network, Sal/Vent Att = salience/ventral attention network, Somatomotor = somatomotor network, Visual = visual network, * = $p_{perm} \leq 0.05$, ** = $p_{perm} \leq 0.01$, *** = $p_{perm} \leq 0.005$, **** = $p_{perm} \leq 0.001$, • = survives multiple-comparisons correction with a false discovery rate of 5%.
